# Male Circumcision and the Epidemic Emergence of HIV-2 in West Africa

**DOI:** 10.1371/journal.pone.0166805

**Published:** 2016-12-07

**Authors:** João Dinis Sousa, Marina Padrão Temudo, Barry Stephen Hewlett, Ricardo Jorge Camacho, Viktor Müller, Anne-Mieke Vandamme

**Affiliations:** 1 KU Leuven—University of Leuven, Department of Microbiology and Immunology, Rega Institute for Medical Research, Clinical and Epidemiological Virology, B-3000, Leuven, Belgium; 2 Center for Global Health and Tropical Medicine, Unidade de Microbiologia Médica, Instituto de Higiene e Medicina Tropical, Universidade Nova de Lisboa, Lisbon, Portugal; 3 Department of Natural Resources, Environment, and Land, CEF, School of Agriculture, University of Lisbon, Lisbon, Portugal; 4 Department of Anthropology, Washington State University Vancouver, Vancouver, Washington, United States of America; 5 Institute of Biology, Eötvös Loránd University, Budapest, Hungary; 6 Parmenides Center for the Conceptual Foundations of Science, Pullach/Munich, Germany; University of Colorado Denver, UNITED STATES

## Abstract

**Background:**

Epidemic HIV-2 (groups A and B) emerged in humans circa 1930–40. Its closest ancestors are SIVsmm infecting sooty mangabeys from southwestern Côte d'Ivoire. The earliest large-scale serological surveys of HIV-2 in West Africa (1985–91) show a patchy spread. Côte d'Ivoire and Guinea-Bissau had the highest prevalence rates by then, and phylogeographical analysis suggests they were the earliest epicenters. Wars and parenteral transmission have been hypothesized to have promoted HIV-2 spread. Male circumcision (MC) is known to correlate negatively with HIV-1 prevalence in Africa, but studies examining this issue for HIV-2 are lacking.

**Methods:**

We reviewed published HIV-2 serosurveys for 30 cities of all West African countries and obtained credible estimates of real prevalence through Bayesian estimation. We estimated past MC rates of 218 West African ethnic groups, based on ethnographic literature and fieldwork. We collected demographic tables specifying the ethnic partition in cities. Uncertainty was incorporated by defining plausible ranges of parameters (e.g. timing of introduction, proportion circumcised). We generated 1,000 sets of past MC rates per city using Latin Hypercube Sampling with different parameter combinations, and explored the correlation between HIV-2 prevalence and estimated MC rate (both logit-transformed) in the 1,000 replicates.

**Results and Conclusions:**

Our survey reveals that, in the early 20^th^ century, MC was far less common and geographically more variable than nowadays. HIV-2 prevalence in 1985–91 and MC rates in 1950 were negatively correlated (Spearman rho = -0.546, IQR: -0.553–-0.546, p≤0.0021). Guinea-Bissau and Côte d'Ivoire cities had markedly lower MC rates. In addition, MC was uncommon in rural southwestern Côte d'Ivoire in 1930.The differential HIV-2 spread in West Africa correlates with different historical MC rates. We suggest HIV-2 only formed early substantial foci in cities with substantial uncircumcised populations. Lack of MC in rural areas exposed to bushmeat may have had a role in successful HIV-2 emergence.

## Introduction

Human Immunodeficiency Virus (HIV) has polyphyletic origins. Type 1 (HIV-1) includes four lineages (groups M, N, O, and P) each separately transmitted to humans from chimpanzees or gorillas [1–2]; Type 2 (HIV-2) covers nine lineages (groups A to I) separately transmitted each time from sooty mangabeys (*Cercocebus atys atys*) [3–8]. HIV-1 group M is responsible for the global pandemic; it is also more pathogenic than HIV-2, and has therefore received much more attention.

HIV-2 has been less studied than HIV-1, but is nevertheless a considerable threat to public health, since it has spread worldwide and is probably infecting more than one million people [9].Of the 9 known HIV-2 groups, only groups A and B have generated widespread epidemics, while all other HIV-2 groups represent either dead-end infections or small clusters [3–8]. Phylogenetic analyses showed that HIV-2 groups A and B started to spread around 1938 (95% CI 1928–47) [10], and 1945 (1931–59) [11], respectively, soon after HIV-1 groups M and O [12–16]. The HIV-2 epicenter has long been recognized to lie in West Africa, particularly in Côte d'Ivoire and Guinea-Bissau, with important foci in other ex-Portuguese colonies [10,11,17–22]. The first large scale sero-epidemiological surveys of HIV-2 started soon after the virus had been discovered, using samples collected between 1985 and 1991 [17–22]. In these surveys, samples from more than 260,000 West African adults without specific risk factors, and from additional tens of thousands commercial sex workers (CSW), patients with sexually transmitted diseases (STD), AIDS, tuberculosis (TB), and hospitalized patients were screened for HIV-2 [17–22]. The surveys covered all 16 countries of the area, several locations per country, and both urban and rural locations and thus they provide a unique snapshot of the HIV-2 situation in West Africa half a century after the epidemic started.

They show a very uneven epidemiological picture. In Côte d'Ivoire the overall urban prevalence (here and in the rest of this article HIV-1/HIV-2 dual infections are subsumed in HIV-2 prevalence) was 2.5%. In Guinea-Bissau it was 7.9%. The urban rates were 1.9% in Gambia, 1.4% in Cape Verde, 1.3% in Burkina Faso, 1.8% in Mali, and <0.8% in the remaining countries [17–22]. Côte d'Ivoire and Guinea-Bissau are the only West African countries with serological evidence of HIV-2 from the 1960s [23–27]. According to a phylogeographical analysis, Guinea-Bissau appears as the probable root of the HIV-2 group A epidemic, but a root in Côte d'Ivoire could not be excluded [10].

Commercial sex work, sexually transmitted diseases, lack of male circumcision (MC), and parenteral transmission promote HIV spread in Africa. Wars and parenteral transmission have been hypothesized to have played a leading role in the early dissemination of HIV-2. Several authors pointed out the independence war of Guinea-Bissau (1963–74) as having potentially given a boost to HIV-2, by increasing sexual [28] and/or parenteral [23,29] transmission. Age cohort effects and data on parenteral exposure have been invoked to support these views [23,28,29].

Evidence for the protective role of MC at the population level has only been published for HIV-1. Two studies [30,31] demonstrated a strong correlation between HIV-1 prevalence in the late 1980s and traditional MC patterns in Africa, as stated in G. P. Murdock's Ethnographic Atlas (EA) [32]. Other studies replicated these findings for most developing countries of the world, using estimates of current MC frequency for many countries [33,34]. Meta-analyses indicate odds ratios of HIV-1 infection for uncircumcised status between 2.5 and 5 [35,36]. A major epidemiological study of HIV-1 and STDs in four African cities examined many potential factors that could explain the differences in HIV-1 prevalence among cities, and MC turned out to be the major factor [37].

Ethnographic atlases and reviews indicate that, in late 19^th^–early 20^th^ century, MC was not traditionally practiced by many West African ethnic groups [32,38–40]. In contrast, modern Demographic and Health Surveys (DHS) [41] show that MC is currently almost universal throughout the region. This discrepancy implies that during the 20^th^ century, a major wave of adoption of MC occurred in West Africa.

Risk factors for HIV-2 positive status have been repeatedly shown to be the same as for HIV-1 positivity: being a CSW [21,42,43], contacts with CSWs [21,28,44], sexual promiscuity, single, or divorced status [24,45], genital ulcer disease [21,43,44,46], lack of MC [21,46,47], transfusions [23,24,42,45], injections [20,29]. Yet, to our knowledge, no study has attempted to associate HIV-2 prevalence with MC frequency. The similarity between the factors contributing to heterosexual transmission of HIV-1 and HIV-2 raises the hypothesis that differences in HIV-2 spread within West Africa may show an association with differences in MC frequency, as has already been established for HIV-1 [30,31,33,34,37].

The scarcity of HIV-2 prevalence data limits the possibilities of testing the association between MC and HIV-2 prevalence. Most UNAIDS reports publish joint HIV prevalence without specifying virus type. Some scientific publications did report the HIV-2 prevalence (or the proportion HIV-2/HIV-1) found in their samples, but they typically used either a countrywide sample, without further regional partition, or focused only on a capital city [48–50]. Besides, these articles provide only sporadic coverage and worked with small sample sizes. The already mentioned serological studies of the period 1985–91 [17–22] constitute a far more complete source, covering dozens of cities of every country, and more than 260,000 samples. Furthermore, they are the oldest data available, and probably carry a footprint of early spread. They are also contemporaneous with similar large scale surveys that were used to calculate the ecological association between MC and HIV-1 prevalence [30,31]. We have therefore focused on these serological data to investigate their correlation with MC, applying Bayesian estimation to infer prevalence rates (see Materials and Methods).

We searched historical patterns of MC in ethnographic databases and literature and complemented this with fieldwork. We used interpolation to estimate MC frequency for each ethnic group over time, between early 20^th^ century and nowadays. We calculated the correlation between MC frequency (with MC calculated at various time points) and HIV-2 seroprevalence in 1985–91 at a city level. We then interpreted these results in the context of the emergence and early spread of HIV-2 groups A and B epidemics.

## Results

### Serology shows uneven geographical distribution of HIV-2

**Fig 1:**
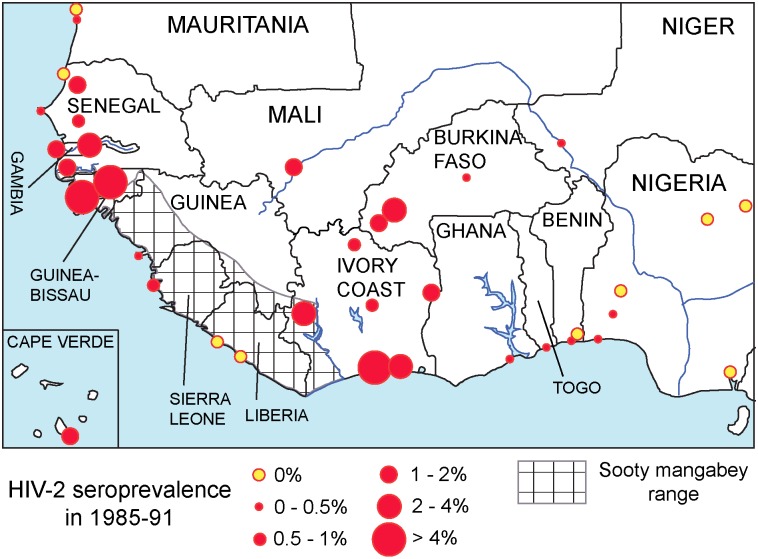
HIV-2 seroprevalence in adults without specific risk factors in West Africa, 1985–91. Complete information on HIV-2 prevalence rates is in S1 Dataset. The checkered area indicates the historical (pre-1950) range of sooty mangabeys [51–53].

HIV-2 serological data of the period 1985–91 meeting our criteria were available for 30 West African cities (listed in Materials and Methods). We applied Bayesian estimation to infer HIV-2 prevalence rates from the sample-based frequencies of HIV-2 seropositives (see Materials and Methods). HIV-2 prevalence was very unevenly distributed, but spots of high prevalence existed in both western and eastern parts of West Africa. Fig 1 depicts the prevalence data and the historical (pre-1950) range of *Cercocebus atys atys* according to primatological sources [51–53].

### Historical male circumcision prevalence has an uneven geographical distribution

To research historical patterns of MC, we first consulted the Ethnologue database and ethnographic maps [54–56]. We then retrieved MC information from major ethnographic atlases and reviews [32,38–40,57,58], and filled in their MC information gaps with primary ethnographic articles and fieldwork in Guinea-Bissau. In total, we covered 218 ethnic groups encompassing almost all West Africa (see Materials and Methods).

MC was far less common, and geographically more patchy in most early (1890–1920) ethnographic accounts than it is in current times. After applying our assumptions, as explained in Materials and Methods, MC was found to be traditionally not practiced or practiced by a minority of men in 72 (33.0%) groups. These results broadly agree with major ethnographic atlases and reviews [32,38–40,57,58], although our coverage of MC permitted to fill gaps in these works. The results are condensed in S2 Dataset, and a concise explanation of the frequency of MC patterns, and its distribution over major ethnic families is in section a) of S1 Text.

Our fieldwork in Guinea-Bissau determined that a pattern of MC in late age persists to these days among non-Muslim groups: among the Balanta, MC was normally done after age 40, though at present it starts to be done between 20 and 30 years of age [59]; among the Manjako and the Felupe MC is done in collective rituals held each ~20 years, which causes many men to remain uncircumcised through adulthood (a pattern seen also among the Jola of the neighboring Casamance region, and among the Yowaba of northern Benin).

### Robust correlation between HIV-2 seroprevalence and historical MC prevalence

**Fig 2:**
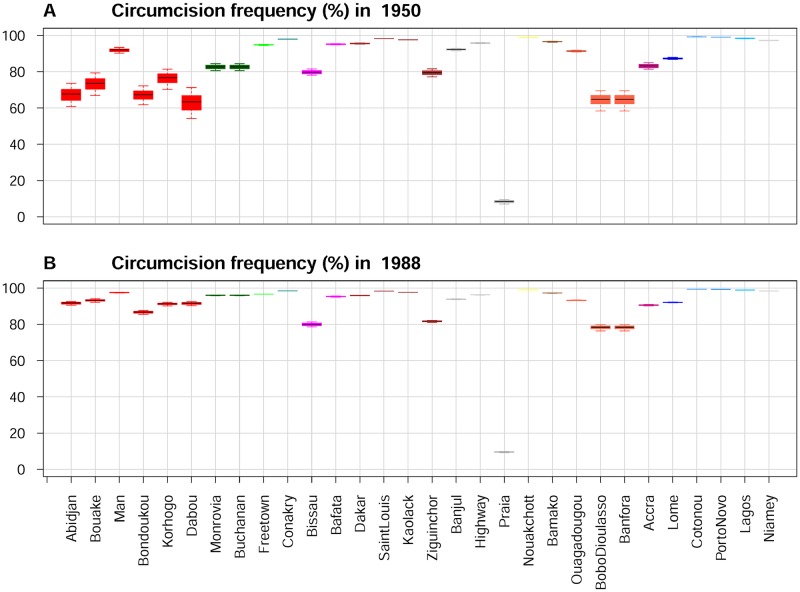
Estimated MC frequencies for the West African cities. Estimated MC frequencies in 1950 (A) and 1988 (B) for the West African cities. Boxes indicate the interquartile range (IQR) of the frequencies; median is indicated by a horizontal line within the box, and whiskers extend to the farthest values that are not more than 1.5 times the IQR away from the box. Cities of the same country appear with the same color. Country colors: Côte d'Ivoire, red; Liberia, dark green; Sierra Leone, green; Guinea, dark cyan; Guinea-Bissau, magenta; Senegal, brown; Gambia, gray; Cape Verde, dark gray; Mauritania, yellow; Mali, chocolate; Burkina Faso, tomato; Ghana, violet-red; Togo, blue; Benin, dodger blue; Nigeria, deep sky blue; Niger, light gray.

We used interpolation to estimate MC frequency for each ethnic group over time, between early 20^th^ century and nowadays (keeping in mind that a major wave of MC adoption occurred for many ethnic groups). Through Latin Hypercube Sampling (LHS) of the parameter values, 1,000 different calculations of estimates of MC frequency per ethnic group were obtained (see Materials and Methods). MC frequencies per city were then compiled based on tables of ethnic partitioning of population (TEPP) derived from censuses and surveys (see Materials and Methods). Fig 2 shows these results for the 30 cities, in 1950 and 1988, under the default model (COLRST-LOGIS).

The correlation between HIV-2 prevalence in 1985–91 and estimated MC in 1988, for our default COLRST-LOGIS model, is substantial and significant (median Spearman rho = -0.560, interquartile range (IQR) = -0.562–-0.560, p≤0.0022 for all comparisons).

We performed two sensitivity analyses. In the first, we used the different models for interpolating, and three different time points. The results were broadly similar across models and time points (Table 1).

**Table 1:** The correlation between HIV-2 prevalence and estimated MC rate at various times and for three models of interpolation.

Model	Time	corr median	corr IQR	p-value median	p-value maximum
COLRST-LOGIS	1950	-0.546	-0.552 –-0.546	0.00181	0.00209
	1970	-0.564	-0.567 –-0.561	0.00117	0.00153
	1988	-0.560	-0.562 –-0.560	0.00130	0.00220
COLRST-LIN	1950	-0.541	-0.546 –-0.541	0.00203	0.00217
	1970	-0.563	-0.566 –-0.557	0.00119	0.00158
	1988	-0.570	-0.570 –-0.564	0.00101	0.00118
COLRSTURB	1950	-0.501	-0.509 –-0.495	0.00477	0.00541
	1970	-0.559	-0.560 –-0.559	0.00133	0.00155
	1988	-0.581	-0.586 –-0.578	0.00075	0.00095

Shown the Spearman rho correlations between HIV-2 prevalence in 1985–91 and estimated MC rate at various times, for all 30 cities included in the study and for three models of MC interpolation. Shown the IQRs of correlations, median and maximum p-values from 1,000 tests with re-sampled parameters.

The independence of the results from the time of MC calculation within the 1950–88 period is not surprising because, even though MC frequency increased rapidly in many ethnic groups during that period, the relative rankings of the ethnic groups (and hence of cities) in MC frequency changed only moderately. Reassuringly, the correlation was similar across all models of MC interpolation.

We then focused on our COLRST-LOGIS default model and performed different calculations with specific subsets of cities (Table 2). First, and following the method of Bongaarts et al. (1989) for HIV-1 [30], we tested the correlation with only the capital/main city of each country included. Second, we tested it also with all main or major cities included.

**Table 2:** The correlation between HIV-2 prevalence and estimated MC rate for different subsets of cities.

Cities included	Num.cities	Time	corr.median	corr.IQR	p-value.median	p-value.maximum
All cities (default)	30	1988	-0.560	-0.562 –-0.560	0.00130	0.00220
Only capital/main cities	16	1988	-0.462	-0.462 –-0.462	0.07177	0.09442
Main and major cities	21	1988	-0.584	-0.591 –-0.584	0.00540	0.00713
Abidjan-connected	20	1960	-0.569	-0.569 –-0.561	0.00889	0.01274

Shown the Spearman rho correlation between HIV-2 prevalence in 1985–91 and estimated MC rate at various times, for different subsets of cities. The cities included in each subset are referred to in S1 Computer Code. Shown the IQRs of correlations, median and maximum p-values from 1,000 tests with re-sampled parameters.

In Central Africa, Kinshasa was the main hub of dissemination of HIV-1 group M [15,60–62]. During the colonial period, Abidjan was, like Kinshasa, the center of a major transportation network, had a strongly male-biased sex ratio, and rampant commercial sex work [63,64]. Both HIV-2 groups A and B are common in Abidjan [50], and these groups and groups G, H, and I, appear to have originated in southwestern Côte d'Ivoire [3,7]. It is thus tempting to hypothesize that Abidjan was the main hub of dissemination of HIV-2. If so, the cities connected to Abidjan by sea, railway, or with known important migration ties to it provide a natural set of cities with relatively homogeneous exposure to HIV-2 exportation from the main hub. We thus calculated the correlation including only these cities, with MC estimated for 1960. The results of these various tests are in Table 2.

The correlations remain significant in all tests done with 20 or more cities (p-values ≤0.013), and in the range -0.46–-0.59. Significance was lost for the smallest dataset with only the main cities; however the correlation was of the same magnitude and the trend was conserved (p-values ≤0.09).

### MC frequency was lower in cities that were probable earlier epicenters based on phylogeographical and serological evidence

**Fig 3:**
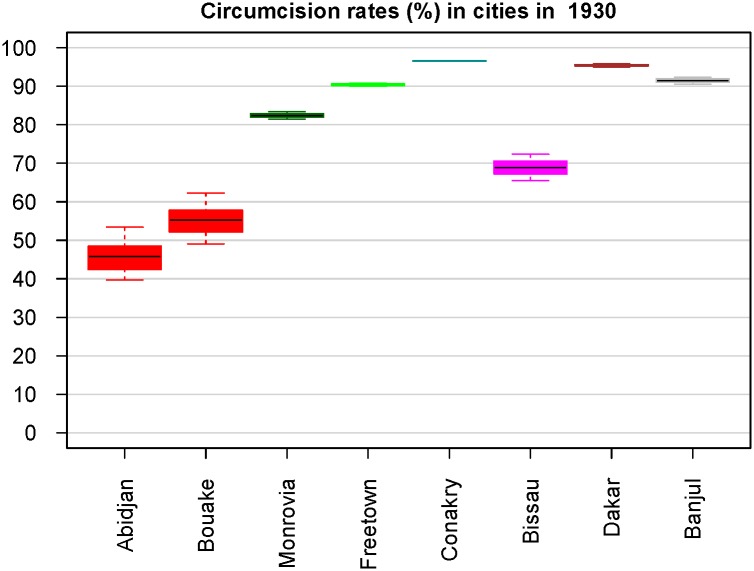
The estimated MC frequencies in 8 large cities at or near the sooty mangabey range in 1930. Cities of the same country appear with the same color. The country colors are the same as in Fig 2 (see Fig 2 legend).

Both HIV-2 groups A and B likely emerged before 1950 [10,11], and the same applies to HIV-1 groups M and O [12–16], suggesting that the probability of HIV epidemic emergence was higher in early 20^th^ century, which may have been related to genital ulcer disease epidemics, injection campaigns, lower historical rates of MC or other factors [61,62,65–67]. The main cities at or near the sooty mangabey range (Dakar, Banjul, Bissau, Conakry, Freetown, Monrovia, Abidjan) were probably exposed to the arrival of rural migrants carrying SIVsmm/HIV-2 infections with the potential to generate an epidemic strain. We thus calculated MC frequencies in these cities in 1930. The results are shown in Fig 3.

The MC rates were markedly lower in the cities of Côte d'Ivoire and Guinea-Bissau, the countries that both serological data [23–27] and phylogeographical analysis [10] suggest were the earliest epicenters.

### The special case of Bissau and the Guinea-Bissau independence war

By the time of the independence war (1963–74), Bissau, with a population near 22,000 in 1960 and 68,000 in 1970 [68,69], received the influx of tens of thousands of Portuguese soldiers (17,000–32,000 in the country throughout the period 1965–74 [70], many of whom frequented Bissau). Many soldiers had contacts with local women and their contacts with high-turnover CSWs were common [24,26]. Therefore, during the war, Bissau had an extremely male-biased sex ratio, rife CSW and, since Europeans are almost universally uncircumcised, a much lowered MC rate (lower than the ones shown in Figs 2 and 3, because these were calculated without considering Europeans).

### MC was not traditionally common in the rural region where epidemic HIV-2 crossed from simians to humans

Our ethnographic literature survey allowed us to map MC throughout West Africa, including in the area inhabited by sooty mangabeys (S2 Dataset). Since phylogenetic analyses of SIVsmm from multiple sites determined that the approximate location of cross-species transmission of five HIV-2 groups (A, B, G, H, and I) was probably southwestern Côte d'Ivoire [3,7], we aimed to compare this region with others of the sooty mangabey range with respect to traditional MC practices.

**Fig 4:**
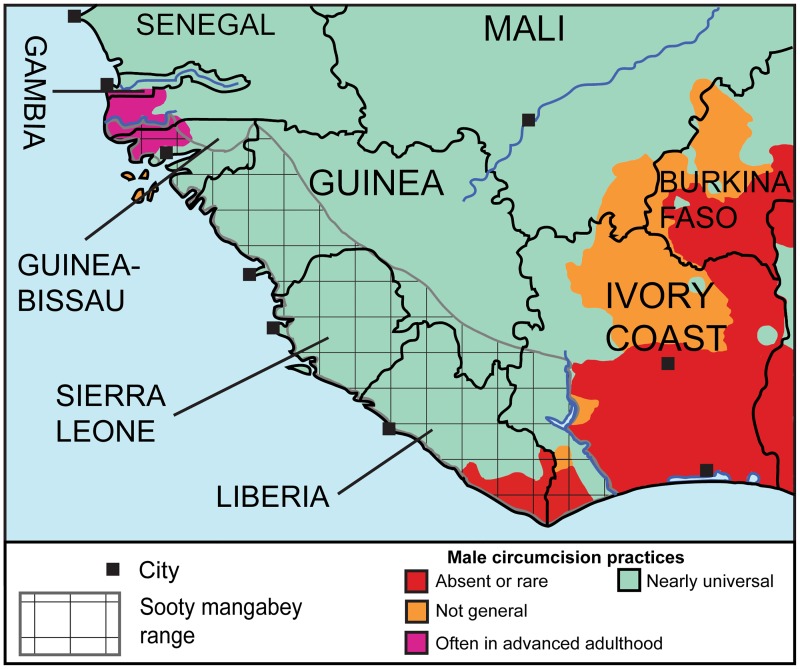
The spread of MC practices at or near the sooty mangabey range in early 20^th^ century. The information supporting this map is in S2 Dataset, with additional explanations in S1 Text. The checkered area indicates the historical (pre-1950) range of sooty mangabeys [51–53].

Focusing on the sooty mangabey range and on MC practices in early 20^th^ century, ethnic groups with absent, uncommon, or not general MC occupy two widely separated areas, one in northern Guinea-Bissau and adjacent Senegal and another in northeast Liberia and southwest Côte d'Ivoire. The latter area contains four ethnic groups in which MC was traditionally absent and still rare in the 1930s, and one group with MC not generally practiced by the 1950s. These groups occupy a contiguous area between the rivers Cess (Liberia) and San Pedro (Côte d'Ivoire) extending northwards up to Tai (Côte d'Ivoire) [54–56,71–76] (Fig 4). The exact ethnic groups involved are described in section b) of S1 Text. This area, which is less than 10% of the sooty mangabey range, encompasses the location where SIVsmm strains phylogenetically close to HIV-2 groups A, B, G, H, and I were found [3,7]. Therefore, the majority of known HIV-2 strains, including the two main ones, may have originated from a sub-area of the sooty mangabey area in which, exceptionally, MC was traditionally absent in the local native ethnic groups [71–74,76].

## Discussion

In this study, we tested the hypothesis of whether the differential HIV-2 spread throughout West Africa could be partly explained by differential male circumcision (MC) rates. To investigate this we first reviewed published data on HIV-2 seroprevalence in the period 1985–91. The HIV-2 serological tests performed in that period were first generation assays, less sensitive and specific than later generation assays, and this might have some confounding effect on our seroprevalence estimates.

Then, we systematically reviewed the ethnographic literature and compiled, to our knowledge, the most comprehensive dataset on MC practices in early 20^th^ century West Africa. The first important finding of our research is that overall MC frequency was far lower and more patchy at the time of early ethnographic observations (1890–1920) than it is today, being either absent or practiced by a minority of men in 33% of the ethnic groups.

In addition, our fieldwork in Guinea-Bissau and literature review identified new areas in Guinea-Bissau and adjacent Senegalese Casamance, where MC was and still is mostly or very often done relatively late in adulthood. This applies to the Balanta, Manjako, and Felupe/Jola ethnic groups. We develop this concisely in section c) of S1 Text.

Our review of the ethnographic literature suggests reasons behind the massive adoption of MC during the 20^th^ century, by peoples traditionally not practicing it: 1) rapid spread of Islam in areas inhabited by non-circumcising peoples (e.g., Gur peoples) [57,77–79]; 2) accelerated ethnic mixing and intermarriage in the cities and labor camps produced by colonialism [80,81]; 3) social pressure to be circumcised to be accepted by women and peers (as documented for Central Africa [82,83]); 4) abandonment of traditional prohibitions of MC (e.g., among the Akan and Ashanti) [84]. Accordingly, several authors refer that, in early 20^th^ century, some not traditionally circumcising groups already had a minority of men circumcised in the towns where they were in contact with circumcising groups [57,58,77,85].

We calculated MC frequencies for ethnic groups and cities over time, and we found, as far as we know for the first time, that HIV-2 prevalence in 1985–91, in West Africa, shows a substantial ecological association with MC, as has been demonstrated for HIV-1, for Africa as a whole, at about the same time period [30,31]. These results lend support to the hypothesis that differential MC has contributed to the differential spread of HIV-2 within West Africa.

Genital ulcer disease (GUD) has a strong co-factor effect in both HIV transmission and acquisition, and uncircumcised men are most at risk of acquiring GUD [86]. Therefore, part of the effect of lack of MC in HIV infection risk is causally mediated by GUD, and GUD may partly mediate the ecological association between HIV-2 prevalence and MC frequency that we have found. However, we could not systematically compare ethnic groups vis à vis GUDs or STDs because ethnographic sources seldom give the relevant detailed accounts.

Differential HIV-2 spread could also reflect viral founder events. Cities with higher HIV-2 prevalence in 1985–91 might have imported the virus earlier, therefore the epidemic focus might have had more time to grow. Indeed, founder events may have been more probable (and might therefore have tended to happen earlier) in cities with increased transmission risk factors, including low MC frequency.

We further calculated MC frequency in 1930, near the time when the two main HIV-2 groups emerged) in the main cities at or near the sooty mangabey range, using roughly contemporaneous tables of ethnic partition of population (TEPP). Abidjan, Bouaké, and Bissau had a strikingly lower MC frequency than the other cities (Fig 3). We cannot exclude biases in the censuses and surveys on which our TEPPs are based, and also the exact ethnic proportions in cities fluctuated with time, probably departing from our time point-specific TEPPs. However, about ¾ of Côte d'Ivoire was populated by non-circumcising ethnic groups (basically those of the main Akan, Lagunaire, Baoulé, Kru, and Gur families; see [56,58,77,78] and S2 Dataset). Therefore, inevitably MC rates were low in its cities, even if the ethnic proportions largely deviated from our TEPPs. In Bissau, the relatively low MC rate was due to the presence of substantial numbers of Cape Verdeans, Balanta, and Manjako [87–89], who either do not practice MC or often practice it in advanced adulthood [34,88,90,91]. Therefore, our calculation of remarkably lower MC rates in Abidjan and Bissau (Fig 3) is a solid result, even if the above mentioned biases exist. Since these are the capital cities of the two countries demonstrably [10,23–27] earlier affected by HIV-2, we can then hypothesize that the formation of the initial epicenters may have been favoured by a low MC rate in certain important cities.

During the independence war, Bissau had a MC rate even lower than depicted in Figs 2 and 3, because of the influx of Portuguese soldiers, and CSW was rife (see Results). These factors may explain: 1) why Guinea-Bissau, while having higher MC frequency than Côte d’Ivoire in mid 20^th^ century (Fig 2), ended up with a higher HIV-2 prevalence decades later; 2) a pattern of acceleration in the HIV-2 epidemic in Guinea-Bissau roughly coinciding with the war, as phylogenetic analysis reveals [11]. Another hypothesis to explain the above facts is that specific parenteral health campaigns may have had a particularly high intensity in Guinea-Bissau compared to other countries and may have accelerated during the war [23,29].

The number of Portuguese soldiers infected by HIV-2 during the war appears to be below 100 [23–26,92]. However, this does not mean they were not relevant. Considering the measured HIV-2 prevalence of 7.5% in Guinea-Bissau in 1985–91, there would be around 30,000 adults infected in the country by then. Applying the exponential growth rate for HIV-2 group A calculated by Lemey et al. (0.201 year^-1^) [11], no more than ~600 Guinea-Bissauans would be infected in 1968 and ~1,800 in 1974. These estimates are compatible with the hypothesis of Portuguese soldiers having played an important role (as core transmitters) in the early establishment of this HIV-2 focus, even if less than 100 became infected.

We studied the spread of MC throughout the sooty mangabey range and found that MC was traditionally absent or rare in a contiguous area encompassing southeastern Liberia and southwestern Côte d'Ivoire. This finding is interesting, considering that this area is less than 10% of the sooty mangabey range (Fig 4) and five HIV-2 groups including the two main ones probably originated there.

It is impossible at this stage to know whether this is just a coincidence or rather that lack of MC helped HIV-2 to emerge from that area. Lack of MC could have facilitated the earliest chains of sexual transmission of SIVsmm between humans. For example, it could have increased the odds of the first human-to-human transmission, between a woman acutely infected by non-adapted SIVsmm after handling bushmeat and her male sexual partner (keeping in mind here that most African butchers and cookers of bushmeat are women, and that these activities may pose a higher risk of SIV infection than hunting itself [93]). It could also have increased the odds of additional sexual transmissions, thus facilitating adaptation by serial transmission [94].

Successful HIV-1 groups (M and O) and HIV-2 groups (A and B) started to spread in early 20^th^ century [10–16], and no successful group emerged after that, despite continued increases of bushmeat hunting intensity, urbanization, and overall injection load. Elsewhere we argued that this temporal pattern could be explained by more intense epidemics of genital ulcer diseases in early 20^th^ century colonial cities [61], or by specific injection campaigns such as those against trypanosomiasis [66,67]. The massive wave of MC adoption by non-circumcising ethnic groups developing over the 20^th^ century, revealed by the present study, could also have contributed to the observed temporal pattern.

In conclusion, we show here, as far as we know for the first time, that HIV-2 prevalence in West Africa, in the period 1985–91, shows a geographical correlation with MC frequency, and that MC frequency was markedly lower in the cities of Côte d'Ivoire and Guinea-Bissau, the only demonstrably old epicenters of HIV-2. These findings reinforce the public health rationale for encouraging voluntary medical MC (VMMC) [95–97], by showing that HIV-1 is not the only retrovirus whose spread may be thwarted or halted by VMMC. It should be kept in mind that HIV-2 already spread well beyond West Africa and is epidemiologically important in countries where MC is uncommon [98,99]. Future studies will be needed to achieve a better understanding of the early epidemiological history of HIV-2.

## Materials and Methods

### HIV-2 prevalence data

We obtained HIV-2 seroprevalence data for 30 cities of 16 West African countries: Abidjan, Bouaké, Man, Bondoukou, Korhogo, Dabou (Côte d'Ivoire), Monrovia, Buchanan (Liberia), Freetown (Sierra Leone), Conakry (Guinea), Bissau, Bafatá (Guinea-Bissau), Dakar, Saint Louis, Kaolack, Ziguinchor (Senegal), Banjul, Highway (Gambia), Praia (Cape Verde), Nouakchott (Mauritania), Bamako (Mali), Ouagadougou, Bobo-Dioulasso, Banfora (Burkina Faso), Accra (Ghana), Lomé (Togo), Cotonou, Porto Novo (Benin), Lagos (Nigeria), Niamey (Niger). The data are described in detail in S1 Dataset.

In compiling HIV-2 seroprevalence data (S1 Dataset), we considered only surveys of adults from the general population (pregnant women, blood donors, professional groups) without known specific risk factors for HIV transmission (excluding data on CSW, hospitalized patients, or patients known to be infected by any STD, HIV or TB) [17–22]. To cover the different countries in an epidemiologically unbiased way, we focused on urban samples, from cities or towns over 20,000 inhabitants at the time of the collection, with more than 200 samples per city (summing up all collections for the city), and with the city well identified. We made a few exceptions from these rules to correct for biases and fill data gaps (described in S1 Dataset). Because low observed frequencies are subject to considerable stochastic error (in particular, zero observed infections might conceal low non-zero seroprevalence), we used Bayesian computation (as implemented in the binom R package (https://cran.r-project.org/web/packages/binom/ [100])) to calculate the posterior distribution and confidence interval of true prevalence values based on the Jeffreys interval method [101], and used the expected value of the distribution as estimated prevalence in the correlation analyses. For Nigeria we considered only the city of Lagos, because seroprevalence was zero in most other locations, reflecting a much later arrival of HIV-2 to this country compared with other countries.

### Estimating male circumcision frequency per ethnic group, both current and in early 20^th^ century

All West African ethnic groups described in the Ethnologue database [54,55] with more than 10,000 people (in the 1990s) were considered. In addition, Kriol groups that do not have a traditional ethnic origin but are numerically important in cities (e.g., Americo-Liberians, Creole-Krio of British West Africa, Brazilian-Beninese, Cape Verdeans) were investigated. Together, our analysis was based on 218 ethno-linguistic and Kriol groups. Published ethnic maps [56] were used as a source for their geographic distribution.

For information about current MC rates, we gathered all DHS (period 2005–2015) [41] from the relevant countries, thus obtaining reliable estimates of MC frequency for each country and, for most countries, for the ethnic groups within the countries. We complemented this information with biomedical papers that reported MC frequencies within the relevant regions. One of us (MPT) performed fieldwork in Guinea-Bissau, based on focus-group discussions and semi-structured interviews (see S1 Appendix).

For information about past MC rates, we used a modified version of the Ethnographic Atlas (EA; Gray (1999) [38]), which expanded on the early work of Murdock (1967) [32] as a starting point for MC data. The EA lacked MC information for many traditional ethnic groups, and for all Kriol groups,which we recovered from major ethnographic reviews [39,40,57,58], early published primary ethnographic field reports, and our fieldwork (S1 Appendix) to complete MC information on the period 1880–1950.

Of the 218 ethno-linguistic and Kriol groups, for 193 (88.5%) we were able to compile information about MC practices (presence/absence, cultural prescription, universality, age of occurrence). For the remaining groups (which had relatively low population numbers), we assumed practices averaged over geographically and culturally (as ascertained by linguistic similarity [54]) very close ethnic groups (see details in S2 Dataset).

### Interpolating male circumcision frequency per ethnic group

We aimed to estimate MC frequency over time, c(t), between the late 19^th^ century and the present, by performing interpolations between the early, ethnography-based estimate, and the current, DHS survey-based estimate. The ethnographic estimates can be considered reliable when they state that MC was either (almost) totally absent or (almost) universal. In intermediate cases, we had to estimate a plausible frequency range based on the verbal account. In our default model, interpolation was based on logistic growth, but we also used other models, as explained below.

For all ethnic groups, we estimated MC frequency, c(t), for the whole period between the years t_min_ = 1851 and t_max_ = 2016. For all groups, there was a survey-based estimate of MC frequency, c_d_, at time t_d_. For many groups, ethnographic sources state that MC was culturally obligatory, universal, and performed before adulthood in early 20^th^ century. These always corresponded to survey-based MC, c_d_>90%. We thus labelled these groups "FIXEDUNIV" and set MC frequency c(t) = c_d_ for the whole t_min_–t_max_ period.

For the remaining, non-FIXEDUNIV groups, MC was either not practiced or not universally practiced in early 20^th^ century. To avoid working with many assumptions, we developed simplified models for MC interpolation, working mostly with parameters and assumptions common to all ethnic groups, and keeping the ethnic group-specific parameters at a minimum.

Hence, for the non-FIXEDUNIV ethnic groups, we defined our default model as follows. We assumed that MC frequency was initially low, and started to increase at time t_1_. For most groups, the parameter t_1_ was set equal to t_1-colrst_, a general parameter sampled from the period 1895–1905. The rationale for this assumption is that this was the time of establishment of colonial rule in all colonies, which prompted rapid and partly enforced mixing between ethnic groups [57,77,78,80,81]. Since several sources suggest that many previously non-circumcising groups were borrowing MC practice from other groups by that time [57,77,78], we assume that the wave of MC adoptions inferred by our preliminary survey (see Introduction) started or greatly accelerated at the start of colonial rule. We call this default scenario the Colonial Rule Start (COLRST) model. For a minority of ethnic groups, for which the ethnographic accounts justified different starting time for MC frequency increase, we used an ethnic group-specific t_1_, rather than the general t_1-colrst_. At time t_1_, we assumed the MC frequency c_1_ to be in a (c_1-low_–c_1-high_) plausibility range. If the ethnographic accounts stated that MC was unknown or not practiced, we assumed this range to be 0–5%, and if they stated that MC existed but was not universal, or practiced late in life (after age 25), we assumed other plausibility ranges based on the ethnographic statements.

Between t_min_ and t_1_, MC frequency was constant at c(t) = c_1_, and then, either: i) increased following a logistic curve saturated at 1, and passing through the point (t_d_, c_d_) (COLRST-LOGIS, our default model); or ii) increased linearly between the points (t_1_, c_1_) and (t_d_, c_d_), and then remained constant until t_max_ (COLRST-LIN model). In the logistic model, we reasoned that any cultural pressures causing adoption of MC will tend to produce universal MC in the long term, and so we used logistic curves saturated at 1 in the distant future, and obtained their parameters analytically as follows.

The logistic progression of MC over time, c(t) is given by:

$$c\left(t\right)=\frac{1}{1+e^{-k\left(t-t_{m}\right)}}$$

For a period t_0_–t_1_, we have estimates of MC frequency at its extremes, c_0_ and c_1_ (either based on ethnographic data or in DHS). Assuming that the logistic growth curve passes by the two points, (t_0_, c_0_) and (t_1_, c_1_), we can write:

$$c_{0}=\;\frac{1}{1+e^{-k\left(t_{0}-t_{m}\right)}}$$

$$c_{1}=\;\frac{1}{1+e^{-k\left(t_{1}-t_{m}\right)}}$$

The logistic parameters, k and t_m_, can then be obtained by solving the above two equation system. The result is:

$$t_{m}=\frac{t_{0}L_{1}-t_{1}L_{0}}{L_{1}-L_{0}}$$

$$k=\frac{L_{1}-L_{0}}{t_{0}-t_{1}}$$

Where $L_{0}=\text{ln}\left(\frac{1}{c_{0}}-1\right)$ and $L_{1}=\text{ln}\left(\frac{1}{c_{1}}-1\right)$

To model a likely acceleration of MC adoption during the urbanization boom (mainly in the period 1950–60), we defined a further Colonial Rule Start and Urbanization (COLRSTURB) model as follows. We used a t_1-colrst_ and a c_1_ as in the previous two models, and a t_2-urb_ (sampled from the 1950–60 range). We calculated the overall implicit exponential growth rate of MC between t_1-colrst_ and t_d_; we assumed that MC grew exponentially by half that rate in the period t_1-colrst_–t_2-urb_, and assumed logistic growth (saturated at 1) in the period t_2-urb_–t_max_.

S1 Fig illustrates the interpolation of c(t) for three ethnic groups, under the three models.

We ran 1,000 different calculations for each of the 3 models described above. For each group, we sampled c_1_ from its plausibility range (c_1-low_–c_1-high_) using Latin Hypercube Sampling (LHS). We performed all our calculations with the R programming environment (http://www.r-project.org/ [102]). All the ethnic group-specific data, estimates, and underlying references, are given in S2 Dataset. The source code is supplied in S1 Computer Code.

### Estimating male circumcision frequency per city

For all the cities included in the study we searched for tables of ethnic partitioning of the population (TEPP) in the demographic (including censuses), historical, or other social science literature, at different moments of the 20^th^ century. We found 24 TEPPs based on census data read from either printed censuses or from articles and books referring them. We found three TEPPs based on published surveys of particular cities, and one based on expert estimates. For several cities no direct city-level TEPP data or estimates were available; in these cases we relied on four tables based on census data of the whole urban population of the administrative division to which the city belonged (Korhogo, Bondoukou, Man, and Dabou); and on two tables based on published estimates for the whole administrative division (Bobo-Dioulasso and Banfora). For Buchanan, Saint Louis, and Nouakchott no tables were available. We assumed for Buchanan a TEPP similar to nearby Monrovia; for Saint Louis, we assumed a TEPP similar to Senegal as a whole, without the Casamance region; for Nouakchott we assumed a TEPP similar to Mauritania as a whole. This information is summarized in S3 Dataset, which lists all relevant TEPPs and HIV-2 prevalence rates. In S4 Dataset this information is detailed per ethnic or regional groups. S2 Text lists the references that support these data. S1 Protocol contains permissions to cite personal communications given by their authors.

For many TEPPs, sources often mentioned broad ethnic or regional categories encompassing several ethnic groups; in such cases we subdivided them into the component ethnic groups following their proportions in the country population. Entries labeled as "Others" were subdivided into all groups belonging to the country and with no direct entry in the TEPP. To accommodate this we built a table of functional regions (corresponding to either entire countries or parts of countries), with aggregates of ethnic groups weighted by their population numbers (S5 Dataset). Entries referring to a foreign nationality were subdivided in all ethnic groups of the referred country proportionally to their numbers. Ethnic proportions in countries were based on the Ethnologue database [55]. For several ethnic groups known to be present in a city at a given time, often no data was given; in such cases we estimated them based on their proportion in the same city at another time or, when using another method of estimation, we justify it explicitly in comments in S4 Dataset.

We estimated MC frequency for the cities at several time points: 1930, 1950, 1970, 1980, and 1988. For each estimation, we used the TEPP nearest in time available for the city, and computed its MC frequency as the average of the MC frequencies of its ethnic groups, weighted by their numbers in the city. We performed 1,000 calculations for each city, time, and calculation mode, based on the ethnic groups' calculations. For each calculation mode and time, we obtained 1,000 calculated values of MC frequency per city.

### Correlation between MC frequency and HIV-2 prevalence

Correlations (Spearman’s rho) were calculated on logit-transformed estimates of MC frequency and HIV-2 prevalence. In a sensitivity analysis, we repeated the correlation tests on several subsets of the cities. As a result of LHS sampling of the parameter values, 1,000 measures of the correlations were obtained for each studied year and interpolation model; we present interquartile ranges (IQR) of the correlation coefficients.

### Ethics statement

The study involved a re-analysis of published HIV prevalence data; individual patient data were not included in the analysis. This research was approved by the Commissie Medische Ethiek of the Katholieke Universiteit Leuven, approval number S59252.

## Supporting Information

S1 Appendix Fieldwork in Guinea-Bissau. (PDF)

S1 Computer Code Source code of the R program used and related files. (ZIP)

S1 Dataset HIV-2 prevalence rates. (XLSX)

S2 Dataset Male circumcision information per ethnic group. (XLSX)

S3 Dataset List of tables of ethnic partition of population of cities. (XLSX)

S4 Dataset Full dataset of tables of ethnic partition of population of cities. (XLSX)

S5 Dataset Dataset of ethnic partition of population of countries and regions. (XLSX)

S1 Fig Progression of MC frequency for 3 ethnic groups over time under 3 models of interpolation (COLRST-LIN, COLRST-LOGIS, and COLRSTURB, see Materials and Methods). (TIF)

S1 Protocol Permissions to cite personal communications. (ZIP)

S1 Text Specific patterns of MC distribution. (PDF)

S2 Text Supplementary references supporting information in Datasets. (PDF)
