# Supplementary material for: Male Circumcision and the Epidemic Emergence of HIV-2 in West Africa
Source: PLoS One. 2016 Dec 7;11(12):e0166805. doi: 10.1371/journal.pone.0166805 (PMC5142780; doi:10.1371/journal.pone.0166805)
Supplement: S1 Appendix — (PDF) [file pone.0166805.s001.pdf]

Male Circumcision and the Epidemic Emergence of HIV-2 in West Africa

*João Dinis Sousa[1,2], Marina Padrão Temudo[3], Barry Stephen Hewlett[4], Ricardo Jorge Camacho[1], Viktor Müller[5,6], Anne-Mieke Vandamme[1,2]

1 KU Leuven - University of Leuven, Department of Microbiology and Immunology, Rega Institute for Medical Research, Clinical and Epidemiological Virology, B-3000 Leuven, Belgium

2 Center for Global Health and Tropical Medicine, Unidade de Microbiologia Médica, Instituto de Higiene e Medicina Tropical, Universidade Nova de Lisboa, Lisbon, Portugal

3 Department of Natural Resources, Environment, and Land, CEF, School of Agriculture, University of Lisbon, Lisbon, Portugal

4 Department of Anthropology, Washington State University Vancouver, Vancouver, USA

5 Institute of Biology, Eötvös Loránd University, Budapest, Hungary

6 Parmenides Center for the Conceptual Foundations of Science, Pullach/Munich, Germany

2016

Supporting Information S1 Appendix: Fieldwork in Guinea-Bissau

During a period of more than two decades MPT has been conducting fieldwork in Guinea-Bissau among the most important ethnic groups. Long-term observation has provided important empirical data about MC practices and their changes across time. Specific research for this article was conducted during 2014 and 2015 in almost 30 villages of the Balanta, Fula, Mandinga, Manjako, Nalu, Beafada, Papel, Mankanya, and Felupe ethnic groups, and also among Cape Verdeans residing in Guinea-Bissau. It consisted of focus-group discussions, semi-structured interviews, informal conversations with key-informants, and village group discussions. For each ethnic group the questions focused on the age at which MC was performed before the start of the independence war and the changes that the practice has been undergoing until the present. In the case of the Balanta, research also aimed to evaluate the findings published by Niang & Boiro (2007) [91]. Cape Verdean descendants living in Bissau and in rural villages were inquired about their own and their socio-cultural group MC practices since the fifties.

The references cited in this Supporting Information can be found in the reference list of the main article.
