# Supplementary figures and images for: Male Circumcision and the Epidemic Emergence of HIV-2 in West Africa
Source: PLoS One. 2016 Dec 7;11(12):e0166805. doi: 10.1371/journal.pone.0166805 (PMC5142780; doi:10.1371/journal.pone.0166805)

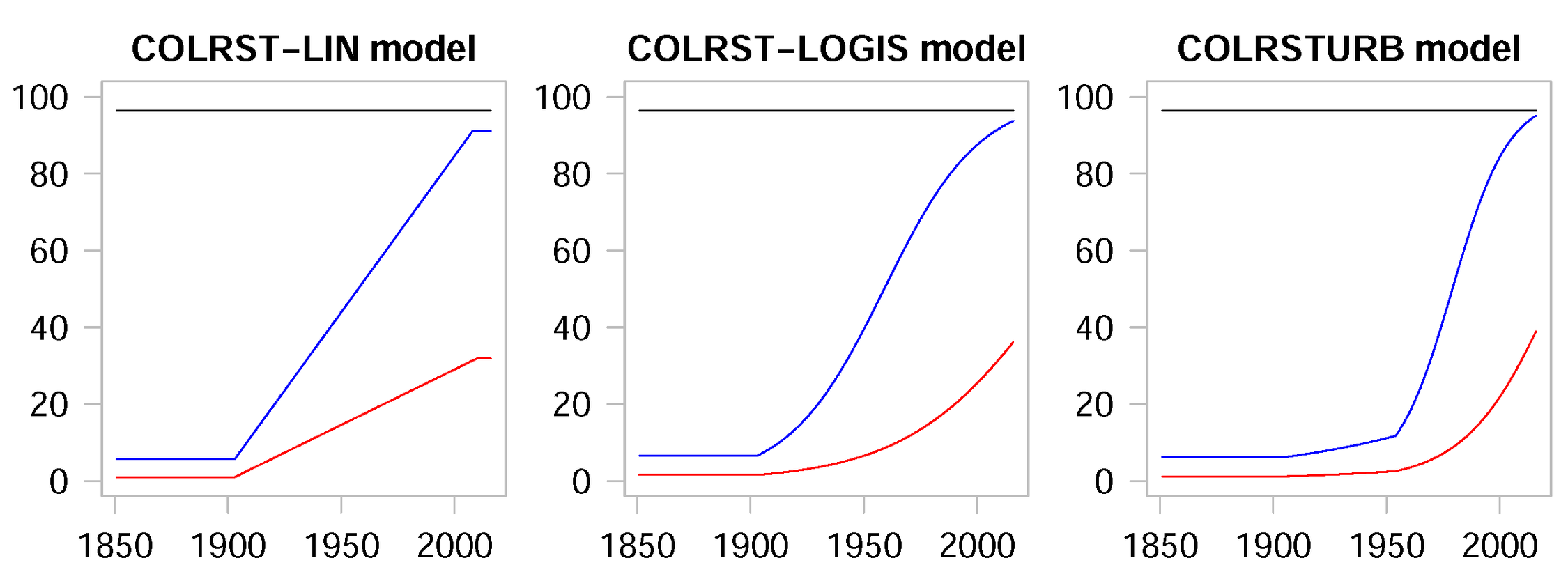

Supplement: S1 Fig — (TIF) [file pone.0166805.s008.tif]
