# Supplementary material for: Male Circumcision and the Epidemic Emergence of HIV-2 in West Africa
Source: PLoS One. 2016 Dec 7;11(12):e0166805. doi: 10.1371/journal.pone.0166805 (PMC5142780; doi:10.1371/journal.pone.0166805)
Supplement: S1 Protocol — (ZIP) [file pone.0166805.s009.zip › PC_MarcAuge.pdf]

X-AntiVirus: PTMail-AV 0.3-0.94.2
X-Scan-Status: AV clean (0.00654 seconds); AS clean (0.02429 seconds)
Date: Tue, 6 Jan 2009 19:18:23 +0100
Subject: Re: Circoncision aux Lagunaires?
From: armand.auge@free.fr
To: Joao Sousa <j.d.sousa@sapo.pt>
X-Mailer: Apple Mail (2.552)

Bien sûr. M.A.
Le mardi, 6 jan 2009, à 18:09 Europe/Paris, Joao Sousa a écrit :

> Bonjour Dr Marc Augé,
>
> Comme conséquence de la nature de ma recherche, je suis encore PLUS interessé à l' absence de circoncision, qu' a la circoncision. Je suis à rechercher le virus HIV-2, et sa propagation differentielle à divers pays de l' Afrique de l' Ouest. La Côte d' Ivoire est unique en Afrique d' Ouest, vis à vis la prevalence que ces virus ont obtenu, et aussi vis à vis la diversité de souches. Mon hypothèse est que l'absence de circoncision (que, on sait maintenant, augmente le risque de transmission heterosexuelle femme->homme), explique cette difference entre la Côte d'Ivoire, et les autres pays. Je suis à preparer une communication pour une conference à Montreál, à Février, sur ce sujet.
>
> J'ai déjà des réferences bibliographiques qu' indiquent absence de circoncision chez les Baoulé, Agni, Bété, et Gourou, mais il me manque à propos des Lagunaires.
>
> Donc, est-ce que je peut dire dans ma communication et dans mon article en préparation, que cette information est une communication personelle de vous? Bien sur qu 'il est mon intention de vous envoier copies de ma communication et article sur ce sujet.
>
> Merci beaucoup!
> Très amicalement
> João
>
> At 08:24 06-01-2009, you wrote:
>> Non. Il n'y a jamais eu de circoncision chez les peuples lagunaires et je ne vois pas pourquoi on trouverait dans la littérature la mention d'une absence. Bien à vous. M.A.
>> Le mardi, 6 jan 2009, à 08:37 Europe/Paris, Joao Sousa a écrit :
