# Supplementary material for: Male Circumcision and the Epidemic Emergence of HIV-2 in West Africa
Source: PLoS One. 2016 Dec 7;11(12):e0166805. doi: 10.1371/journal.pone.0166805 (PMC5142780; doi:10.1371/journal.pone.0166805)
Supplement: S1 Text — (PDF) [file pone.0166805.s010.pdf]

Male Circumcision and the Epidemic Emergence of HIV-2 in West Africa

*João Dinis Sousa[1,2], Marina Padrão Temudo[3], Barry Stephen Hewlett[4], Ricardo Jorge Camacho[1], Viktor Müller[5,6], Anne-Mieke Vandamme[1,2]

1 KU Leuven - University of Leuven, Department of Microbiology and Immunology, Rega Institute for Medical Research, Clinical and Epidemiological Virology, B-3000 Leuven, Belgium

2 Center for Global Health and Tropical Medicine, Unidade de Microbiologia Médica, Instituto de Higiene e Medicina Tropical, Universidade Nova de Lisboa, Lisbon, Portugal

3 Department of Natural Resources, Environment, and Land, CEF, School of Agriculture, University of Lisbon, Lisbon, Portugal

4 Department of Anthropology, Washington State University Vancouver, Vancouver, USA

5 Institute of Biology, Eötvös Loránd University, Budapest, Hungary

6 Parmenides Center for the Conceptual Foundations of Science, Pullach/Munich, Germany

2016

Supporting Information S1 Text: Specific patterns of MC distribution

a) Summary of findings about Male Circumcision (MC) distribution

Our analysis covered 218 ethnic groups (including Kriol groups) and shows that, in early 20th century, MC was: 1) culturally mandatory and done before adulthood in 125/218 (57.3%) ethnic groups; 2) not practiced (sometimes even forbidden), or practiced by a minority of men in 72 (33.0%) groups; 3) practiced in advanced adult age (over 25) by many or most men, in at least 5 (2.3%) and perhaps 6 (2.8%) groups; 4) practiced but not universally, with the available information not sufficient to determine whether it was in a minority or a majority of men, in the remaining 15 (6.9%) groups. The references supporting these results are in **S2 Dataset** and **S2 Text**.

Large regions and ethnic group families traditionally lacking MC practice were identified: 1) several groups of the Kru family in southeastern Liberia and southwestern and south-central Côte d'Ivoire; 2) all Lagunaire groups of southeastern Côte d'Ivoire; 3) almost all groups of the Akan family in southeastern and central Côte d'Ivoire and southwestern and central Ghana; 4)

most groups of the Gur family in the highlands of northeastern and north-central Côte d'Ivoire, southern Burkina-Faso, and northern parts of Ghana, Togo, and Benin. These results broadly agree with major ethnographic atlases and reviews (Murdock [32,38], Baumann & Westermann [58], Frobenius [39], Jensen [40]), although our coverage of MC permitted to fill gaps in these works (**S2 Dataset**).

b) MC patterns in southeastern Liberia and southwestern Côte d'Ivoire

The Kru-Klao and Grebo ethnic groups not only did not practice MC traditionally [71–76] but even "abhorred" it [76]; this applied to most or all Grebo subgroups, such as the Palepo (often called "Half Grebo"), Tepo, Pye, and Plapo (often called "Krumen") [71,72]. MC was still rare in their villages in the 1950s-1960s, although in some communities medical doctors and the cultural influence of other ethnic groups had already brought MC [75,76]. Today, the DHS indicate nearly universal MC in the area [41]. Among the Glio-Oubi of the Tai forest, MC was practiced in the 1950s, but it was not general, and showed signs of having been adopted recently [75]. In addition, other groups of the Kru family adjacent to this area (Krahn, Sapo, and possibly Guéré, and Bakwé) already practiced MC in early 20th century [38,72] but may have adopted it soon before that [75,76].

c) MC performed late in age in northern Guinea-Bissau and Casamance

The Balanta are the biggest ethnic group in Guinea-Bissau and although their homeland is located in the north, they started to migrate to the South in search of rice fields since 1915, also becoming the dominant group there. Initiation into manhood rituals was usually conducted every 4–6 years, but the age at which a man was allowed to do it was (and still is) decided by the head of the compound and is dependent upon the compound's human and economic resources [59]. This meant that most men usually did MC above 40 years old. Nonetheless, young men's fight to achieve full adulhood at a younger age triggered a recent change and most start to do MC between 20–30 years old together with other men over 40 (there are still men over 60 who are still uncircumcised). Our findings contradict the ones of Niang & Boiro (2007) [91] who state that the Balanta perform two MC (respectively named *Foo Ntiufa* and *Foo Garandi*), the first one being only a small incision made on the foreskin to men aged between 18–20 years old. Indeed, *Foo Ntiufa* is conducted only in around 10 among the hundreds of Balanta villages of Guinea-Bissau and, according to our interviewees, no incisions are performed. Contrary to the Balanta, among the Manjako and the Felupe men are allowed to perform MC early than 20 years old. If they miss the ritual when they are that age, they then have to wait

sometimes more than 20 years to do it, as rituals only occur every 20–25 years [90].

Among the Fula, Mandinga, Beafada and Nalu, MC used to be practiced when around 15–18 years old. After Independence of the country, the age at which boys do initiation into manhood among these Muslim groups has been progressively reduced and, at present, many practice MC between 3–10 years old. The case of Cape Verdeans is different: while in their homeland MC is not practiced at all, when they live or were born in Guinea-Bissau the adoption of this practice becomes an individual decision – as to be called "*blufu*" (non-circumcised) is an insult. According to our interviewees, between the fifties and early seventies, the Cape Verdean migrants were not circumcised. However, some of their sons used to run away from home and joined their peers of other ethnic groups in the male initiation barracks. Others, would perform MC at an hospital when they were around 18 and the rest would never practice MC.

The references cited in this Supporting Information can be found in the reference list of the main article.
