# Supplementary material for: Male Circumcision and the Epidemic Emergence of HIV-2 in West Africa
Source: PLoS One. 2016 Dec 7;11(12):e0166805. doi: 10.1371/journal.pone.0166805 (PMC5142780; doi:10.1371/journal.pone.0166805)
Supplement: S2 Text — (PDF) [file pone.0166805.s011.pdf]

Male Circumcision and the Epidemic Emergence of HIV-2 in West Africa

*João Dinis Sousa[1,2], Marina Padrão Temudo[3], Barry Stephen Hewlett[4], Ricardo Jorge Camacho[1], Viktor Müller[5,6], Anne-Mieke Vandamme[1,2]

1 KU Leuven - University of Leuven, Department of Microbiology and Immunology, Rega Institute for Medical Research, Clinical and Epidemiological Virology, B-3000 Leuven, Belgium

2 Center for Global Health and Tropical Medicine, Unidade de Microbiologia Médica, Instituto de Higiene e Medicina Tropical, Universidade Nova de Lisboa, Lisbon, Portugal

3 Department of Natural Resources, Environment, and Land, CEF, School of Agriculture, University of Lisbon, Lisbon, Portugal

4 Department of Anthropology, Washington State University Vancouver, Vancouver, USA

5 Institute of Biology, Eötvös Loránd University, Budapest, Hungary

6 Parmenides Center for the Conceptual Foundations of Science, Pullach/Munich, Germany

2016

Supporting Information S2 Text: Supplementary references supporting information in Datasets

This Supporting Information contains supplementary reference lists of the references that support the Datasets used in the computation.

<u>INDEX</u>

Part I – References of the Dataset of HIV-2 prevalence rates (S1 Dataset)

Part II – References of the Dataset of male circumcision information per ethnic group (S2 Dataset)

Part III – References of the Datasets of tables of ethnic partition of population of cities and regions (S4 Dataset and S5 Dataset)

Part IV – Personal Communications (S2 Dataset)

Part I – References of the Dataset of HIV-2 prevalence rates

Adu-Sarkodie Y, Tetteh C, Appiah-Denkyira E (1996) Infrequent Occurrence of HIV-2 in Blood Donors and Pregnant Women in a Rural Community in Ghana. Sex Transm Dis 23: 526–527.

Andreasson PA, Dias F, Teixeira Goudiaby JM, Naucler A, Biberfeld G (1989). HIV-2 infection in prenatal women in Guinea-Bissau. 5th International Conference on AIDS. Jun 4–9, 1989. 5: 1000, abstract no. M.G.P.16.

Brun-Vézinet F, Delaporte E, Simon F, Dazza MC, Peeters M, et al. (1988) Épidemiologie de l'Infection HIV-2 en Afrique. Médecine et Maladies Infectieuses 18: 698–701.

Chen Z, Luckay A, Sodora DL, Telfer P, Reed P, et al. (1997) Human Immunodeficiency Virus Type 2 (HIV-2) Seroprevalence and Characterization of a Distinct HIV-2 Genetic Subtype from the Natural Range of Simian Immunodeficiency Virus-Infected Sooty Mangabeys. J Virol 71: 3953–3960.

De Cock KM, Brun-Vézinet F (1989) Epidemiology of HIV-2 infection. AIDS 3(Suppl 1): S89–S95.

De Cock KM, Brun-Vézinet F, Soro B (1991) HIV-1 and HIV-2 infections and AIDS in West Africa. AIDS 5(Suppl 1): S21–S28.

Djomand G, Greenberg AE, Sassan-Morokro M, Tossou O, Diallo MO, et al. (1995) The Epidemic of HIV/AIDS in Abidjan, Côte d'Ivoire: A Review of Data Collected by Project RETRO-CI from 1987 to 1993. J Acquir Immune Defic Syndr 10: 358–365.

Gao F, Yue L, White AT, Pappas PG, Barchue J, et al. (1992) Human infection by genetically diverse SIV_{SM}-related HIV-2 in West Africa. Nature 358: 495–499.

Harry TO, Ekenna O, Chikwem JO, Mohammed I, Sakwa M, et al. (1993) Seroepidemiology of Human Immunodeficiency Virus Infection in Borno State of Nigeria by Sentinel Surveillance. J Acquir Immune Def Syndr 6: 99–103.

Kosia A, Stevens T, Kargbo A (1993) Epidemiology of HIV-2 in Sierra Leone. 9th International Conference on AIDS, June 6-11, 1993. 9: 676, abstract no. PO-C07-2752.

Lahmeyer J (2006) Population Statistics: historical demography of all countries, their divisions and towns. Available: http://www.populstat.info .

Lakiss S, Kourouma K, Diallo MP, Sabbatani S, Rezza G, et al. (1991) HIV-1/2 seroprevalence in Guinea Conakry. 7th International Conference on AIDS, June 16-21, 1991. 7: 373, abstract no. M.C.3300.

Maiga MA, Turcotte F, Doucouré A, Sanogo B, Sidibé D, et al. (1992) Séroprévalence des anticorps contre le virus de l'immunodeficience humaine (VIH) chez les femmes enceintes de Bamako et de Sélingué (Mali). Médecine d'Afrique Noire 39: 94–98.

Mohammed I, Nasidi A, Chikwem JO, Williams EE, Harry TO, et al. (1988) HIV infection in Nigeria. AIDS 2: 61–64.

Neequaye AR, Neequaye JE, Biggar RJ, Mingle JAA, Drummond J, et al. (1997) HIV-1 and HIV-2 in Ghana, West Africa: Community surveys compared to surveys of pregnant women. West Afr J Med 16: 102–108.

Oelman B, Wilkins HA, Hughes A, Whittle H, Jaiteh KO, et al. (1989) The epidemiology of HIV1 and HIV2 in the Gambia, West Africa. 5th International Conference on AIDS, June 4-9, 1989. 5: 1008, abstract no. T.G.P.30.

Olaleye OD, Bernstein L, Ekweozor CC, Sheng Z, Omilabu SA, et al. (1993) Prevalence of Human Immunodeficiency Virus Types 1 and 2 in Nigeria. J Inf Dis 167: 710–714.

Rémy G (1993). Image Géographique des Infections à VIH en Afrique de l'Ouest: Faits et Interrogations. Médecine d'Afrique Noire 40(1): 15–21; 40(2): 81–95; 40(3): 161–168.

Rodier MH, Berthonneau J, Bourgoin A, Giraudeau G, Agius G, et al. (1995) Seroprevalences of toxoplasma, malaria, rubella, cytomegalovirus, HIV and treponemal infections among pregnant women in Cotonou, Republic of Benin. Acta Tropica 59: 271–277.

Sangaré L, Meda N, Lankoandé S, Van Dyck E, Cartoux M, et al. (1997) HIV infection among pregnant women in Burkina Faso: a nationwide survey. Int J STD & AIDS 8: 646–651.

Schim van der Loeff MF, Aaby P (1999) Towards a better understanding of the epidemiology of HIV-2. AIDS 13(Suppl A): S69–S84.

Schim van der Loeff MF (2003) HIV-2 in West Africa. Epidemiological studies. University of Amsterdam, Thesis Dissertation, FileID 69013, http://dara.uva.nl/document/69013 .

Schim van der Loeff MF, Sarge-Njie R, Ceesay S, Awasana AA, Jaye P, et al. (2003) Regional differences in HIV trends in The Gambia: results from sentinel surveillance among pregnant women. AIDS 17: 1841–1846.

United States Bureau of the Census (1993) Recent HIV seroprevalence levels by country: June 1993. Washington DC: Health Studies Branch, Center for International Research, US Bureau of the Census, Research Note no. 9.

United States Bureau of the Census (1996) Recent HIV seroprevalence levels by country: June 1996. Washington DC: Health Studies Branch, International Programs Center, US Bureau of the Census, Research Note no. 21.

Wilkins A, Hayes R, Alonso P, Baldeh S, Berry N, et al. (1991) Risk factors for HIV-2 infection in The Gambia. AIDS 5: 1127–1132.

Williams EE, Mohammed I, Chikwem JO, Akinsete R, Udofia O, et al. (1990) HIV-1 and HIV-2 antibodies in Nigerian populations with high- and low-risk behaviour patterns. AIDS 4: 1041–1042.

Part II – References of the Dataset of male circumcision information per ethnic group

Almeida A (1947) Da medicina gentílica dos Bijagós. In: Conferência Internacional dos Africanistas Orientais, Bissau, 1947, Volume V. Lisboa: Junta de Investigações Coloniais.

Ames D (1959) Belief in 'Witches' among the Rural Wolof of the Gambia. Africa: J Int Afr Inst 29: 263–273.

Andrade E (1998) Do Mito à História. In: Veiga M (Editor) Cabo Verde: Insularidade e Literatura. Paris: Éditions Karthala.

Arcin A (1907) La Guinée française: races, religions, coutumes, production, commerce. Paris: Augustin Challamel.

Augé M (1975) Théorie des pouvoirs et idéologie: étude de cas en Côte d'Ivoire. Paris: Hermann.

Bangré H (2004) Bénin : la circoncision à partir de 28 ans pour devenir adulte. Afrik.com, December 16, 2004. Available: http://www.afrik.com/article7963.html

Banton M (1957) West African city: a study of tribal life in Freetown. London: Oxford University Press.

Bassir O (1954) Rites among the Aku (Yoruba) of Freetown. Africa: J Int Afr Inst 24: 251–256.

Baumann H, Westermann D (1962) Les peuples et les civilizations de l'Afrique. Paris: Payot.

Bernard SJ (1965) EHESSStructures et relations sociales en pays Bisa (Haute-Volta). Cahiers d'Études Africaines 5: 161–247.

Binet E (1900) Observations sur les Dahoméens. Bull Soc Anthrop Paris 5(1): 244–253.

Biyi E (1929–30) The Kru and related peoples, West Africa. J Roy Afr Soc 29: 71–77 (1929); 29: 181–188 (1930).

Bouche PB (1885) Sept ans en Afrique occidentale : la côte des esclaves et le Dahomey. Paris: Plon, Nourrit, et Cie.

Brooks GE (1984) The Observance of All Souls' Day in the Guinea-Bissau Region: A Christian Holy Day, an African Harvest Festival, an African New Year's Celebration, or All of the above(?). History in Africa 11: 1–34.

Brooks GE (2003) Eurafricans in Western Africa: Commerce, Social Status, Gender, and Religious Observance From the Sixteenth to Eighteenth Century. Athens, OH: Ohio University Press.

Bruel G (1935) La France Équatoriale Africaine: le pays, les habitants, la colonization, les pouvoirs publics. Paris: Larose Éditeur.

Brukum NJK (1997) The Northern Territories of the Gold Coast under British Colonial Rule, 1897–1956: A Study in Political Change. University of Toronto, Thesis Dissertation, ISBN 0612282724–9780612282728.

Brunet L, Giethlen L (1900) Dahomey et dépendances, historique général, organisation, administration, ethnographie, productions, agriculture, commerce. Paris: Augustin Challamel.

Bryk F (1934) Circumcision in Man and Woman: its History, Psychology, and Ethnology. Honolulu: Univ Press of the Pacific.

Bugiel V (1922) Une contribution à l'ethnographie mandingue. Bulletins et Mémoires de la Société d'Anthropologie de Paris VII(3): 91–114.

Cammilleri S (2010) A identidade cultural do povo Balanta. Lisboa: Edições Colibri.

Cardinall AW (1920) The Natives of the Northern Territories of the Gold Coast: Their Customs, Religion and Folklore. London: George Routledge and Sons.

Carreira A (1961) Mutilações étnicas nos Manjacos. Boletim Cultural da Guiné Portuguesa 16: 83–102.

Carreira A (1961b) Símbolos, ritualistas, e ritualismos animo-fetichistas na Guiné Portuguesa. Boletim Cultural da Guiné Portuguesa 16: 505–540.

Carreira A (1961c) Organização social e económica dos povos da Guiné Portuguesa. Boletim Cultural da Guiné Portuguesa 16: 641–736.

Carreira A (1962) Guiné Portuguesa: população autóctone segundo os recenseamentos para fins fiscais. Bissau: Imprensa Portuguesa.

Carvalho Viegas LA (1936–40) Guiné Portuguesa [Vol I: 1936; Vol II: 1939; Vol III: 1939–40]. Lisboa: Ministério do Ultramar e Colónia Portuguesa da Guiné.

Chéron G (1933) La circoncision et l'excision chez les Malinké. Journal de la Société des Africanistes 3: 297–303.

Clarke R (1863) Sketches of the Colony of Sierra Leone and Its Inhabitants. Trans Ethnol Soc London 2: 320–363.

Clozel FJ, Villamur R (1902) Les Coutumes Indigènes de la Côte d'Ivoire. Paris: Augustin Challamel.

Corre A (1888) Les peuples du Rio Nunez (côte occidentale d'Afrique). Mémoires de la Société d'Anthropologie de Paris 30 (2éme série): 42–73.

Cruickshanck B (1853) Eighteen years on the Gold Coast of Africa, including an account of the native tribes, and their intercourse with Europeans. London: Hurst and Blackett.

Daniell WF (1849) Sketches of the medical topography and native diseases of the Gulf of Guinea, western Africa. London: Samuel Highley.

Daniell WF (1856) On the Ethnography of Akkrah and Adampe, Gold Coast, Western Africa. J Ethnol Soc London 4: 1–32.

Dantas Pereira J (1996) Pontas e ponteiros na Guiné-Bissau. Actas da II Reunião Internacional de História de África 2: 181–185.

Daumas E (1864) Moeurs et coutumes de l'Algérie. Paris: Librairie de L. Hachette et Cie.

D'Azevedo WL (1962) Some Historical Problems in the Delineation of a Central West Atlantic Region. Ann New York Acad Sci 96: 512–538.

Delacour A (1912–13) Les Tenda (Koniagui, Bassari, Badyaranké) de la Guinée Française. Revue Ethnogr Sociol 3: 287–296; 370–381 (1912); 4: 31–52; 105–120; 140–153 (1913).

Delafosse M (1893) Les Agni (Pai-Pi-Bri). L’Anthropologie 4: 402-45.

Delafosse M (1912) Haut-Sénégal-Niger: le pays, les peuples, les langues. Paris: Larose.

Delafosse M, Poutrin L (1930) Enquête coloniale dans l'Afrique Française Occidentale et Équatoriale, sur l'organization de la famille indigène, les fiançailles, le mariage, avec une esquisse générale des langues de l'Afrique. Paris: Soc d'Éditions Géographiques, Maritimes et Coloniales.

Delval R (1980) Les Musulmans au Togo. Paris: Centre de Hautes Études sur l'Afrique et l'Asie Modernes.

The DHS Program (2015). Demographic and Health Surveys. Calverton, Maryland: ICF International. Available: http://dhsprogram.com

Dieste JLM (2013) Health and Ritual in Morocco: Conception of the Body and Healing Practices. Leiden: Koninklijke Brill.

Dieterlen G, Ligers Z (1963) Notes sur les tambours-de-calebasse en Afrique occidentale. Journal de la Société des Africanistes 33: 255–274.

Dorjahn VR (1959) The Organization and Functions of the "Ragbenle" Society of the Temne. Africa: J Int Afr Inst 29: 156–170.

Dotse AK (2011) The origins and brief history of the Ewe people. XXXX Publications. Available: http://www.bakbikbook.com/detail-pdf/the-origins-and-brief-history-of-the-ewe-people-523447/

Drain PK, Smith JS, Hughes JP, Halperin DT, Holmes KK (2004) Correlates of National HIV Seroprevalence: an Ecologic Analysis of 122 Developing Countries. J Acquir Immune Defic Syndr 35: 407–420.

Drain PK, Halperin DT, Hughes JP, Klausner JD, Bailey RC (2006) Male circumcision, religion, and infectious diseases: an ecologic analysis of 118 developing countries. BMC Infect Dis 6: 172.

Duchemin GJ (1947) L'organization religieuse et son rôle politique dans le royaume Sérére du Sine (Sénégal). In: Conferência Internacional dos Africanistas Orientais, Bissau, 1947, Volume V. Lisboa: Junta de Investigações Coloniais.

Dumsmuir WD, Gordon EM (1999) The history of circumcision. BJU Int 83(Suppl 1): 1–12.

Dupire M, De Tressan M (1955) Devinettes peules et Bororo. Africa: J Int Afr Inst 25: 375–392.

Dupire M, Boutillier JL (1958) Le pays Adioukrou et sa palmeraie (Basse Côte d'Ivoire): Étude socio-économique. Paris: Office de la Recherche Scientifique et Technique Outre-Mer.

Échard N (1969) Histoire du peuplement : les traditions orales d'un village sudye, Shat (Filingué, République du Niger). Journal de la Société des Africanistes 39: 57–78.

Foà E (1895) Le Dahomey. Paris: A. Hennuyer.

Frazer JG (1938) Native races of Africa and Madagascar. London: Percy Lund Humphries & Co.

Frobenius L, Von Wilm R (1929) Atlas Africanus: Belege zur Morphologie der afrikanischen Kulturen. München: C H Beck.

Froelich JC (1955) Carte des populations de l'Afrique Noire. Paris: Sécretariat Général du Gouvernement, Direction de la Documentation.

Froelich JC (1959) Notes sur les Mboum du Nord-Cameroun. Journal de la Société des Africanistes 29: 91–117.

Froelich JC, Alexandre P, Cornevin R (1963) Les populations du Nord-Togo. Paris: Presses Universitaires de France.

Froelich JC (1968) Les montagnards "Paléonigritiques". Paris: ORSTOM.

Gausset Q (1999) Islam or Christianity? The Choices of the Wawa and the Kwanja of Cameroon. Africa: J Int Afr Inst 69: 257–278.

Gauthier J, Wangermez J (1964) Caractères et variations morphologiques des Fali du Tinguelin, Nord-Cameroun. Bulletins et Mémoires de la Société d'anthropologie de Paris. XI Série, 6: 585–610.

Gayibor NL (1991) Histoire du Petit-Popo et du royaume Guin. Lomé: Éditions Haho and ORSTOM.

Glazier SD (Editor) (2001) Encyclopedia of African and African-American Religions. New York: Routledge.

Gomes Barbosa OC (1946) Breve Notícia dos Costumes Étnicos dos Indígenas da Tribo Biafada. Boletim Cultural da Guiné Portuguesa 1: 205–271.

Gordon J (1953) Some Oral Traditions of Denkyira. Transactions of the Gold Coast and Togoland Historical Society 1: 27–33.

Gray JP, editor (1999) Ethnographic Atlas Revised by World Cultures. New York: York College, CUNY.

Griaule M (1956) Note sur le couteau de circoncision Bozo. Journal de la Société des Africanistes 26: 7–8.

Grindal BT (1972) An Ethnographic Classification of the Sisala of Northern Ghana. Ethnology 11: 409–424.

Gubry P (1990). Aspects contemporains de la mortalité au Cameroun septentrional. Conference Groupe Mega-Tchad sur la mort dans le bassin du Lac Tchad, approches multidisciplinaires, Paris, September 12-14, 1990.

Hawthorne W (2001) Nourishing a stateless society during the slave trade: the rise of Balanta paddy-rice production in Guinea-Bissau. J Afr Hist 42: 1–24.

Henric (1902) Notes d'ethnographie et d'anthropologie sur les Haoussas (Afrique Centrale). Annales d'hygiène et de médecine coloniales 5: 414–431.

Hewlett BS, Barnett D (No Date). Unpublished circumcision data extracted from unpublished ethnographic survey notes of African cultures conducted by George Peter Murdock before 1959.

Holas B (1957) L'Évolution du Schéma Initiatique chez les Femmes Oubi (Région de Täi, Côte d'Ivoire). Africa: J Int Afr Inst 27: 241–250.

Holmgren B, da Silva Z, Larsen O, Vastrup P, Andersson S, et al. (2003) Dual infections with HIV-1, HIV-2 and HTLV-I are more common in older women than in men in Guinea-Bissau. AIDS 17: 241–253.

Huttel W (1953) Contribution à l'Anthropologie du Noir d'Afrique. Acta Tropica 10(2): 134–140; 10(3): 251–258.

IRIN (2006) Guiné-Bissau: Circuncisão masculina pode reduzir o risco de infecção por HIV. IRIN News: Humanitarian News and Analysis, Febr 23, 2006. Available: http://www.irinnews.org/report/46085/

Jensen AE (1933) Beschneidung und Reifezeremonien bei Natürvolker. Frankfurt a.M.: Veröffentlichung des Forschungsinstituts für Kulturmorphologie.

Joseph G (1910) Notes sur les Avikams de la lagune de Lahou et les Didas de la région du Bas-Bandama. Bulletins et Mémoires de la Société d'anthropologie de Paris, VI Série, 1: 234–247.

Klose H (1899) Togo unter deutscher Flagge: Reisebilder und Beobachtungen. Berlin: Dietrich Reimer.

Köbben A (1956) Études Éburnéennes; Le Planteur Noir. Abidjan: Inst Français d'Afrique Noire, Centre de Côte d'Ivoire.

Kole NAM (1955) The Historical Background to Krobo Customs. Transactions of the Gold Coast and Togoland Historical Society 1: 133–140.

Krämer (1906) Anthropologische Notizen über die Bevölkerung von Sierra Leone. Globus: illustrierte Zeitschrift für Länder- und Völkerkunde 90: 13–17.

Kurtz (1969) Ethnographic Survey of Southeastern Liberia: The Grebo Speaking Peoples. Monrovia: Tubman Center of African Culture.

Labouret H (1914) Notes contributives a l'étude du peuple Baoulé. Revue Ethnogr Sociol 5: 83–91; 181–94.

Lagarde E, Schim van der Loeff M, Enel C, Holmgren B, Dray-Spira R, et al. (2003) Mobility and the spread of human immunodeficiency virus into rural areas of West Africa. Int J Epidemiol 32: 744–752.

Langley ER (1932) The Kono People of Sierra Leone. Their Clans and Names. Africa: J Int Afr Inst 5: 61–67.

Lapidus IM (1988) A History of Islamic Societies. Cambridge: Cambridge University Press.

Lebeuf JP (1938) La circoncision chez les Kotoko dans l'ancien pays Sao. Journal de la Société des Africanistes 8: 1–9.

Leiris M, Schaeffner A (1936) Les rites de circoncision chez les Dogon de Sanga. Journal de la Société des Africanistes 6: 141–161.

Lestrange M (1950) Contribution à l'étude de l'anthropologie des Noirs d'A.O.F. — II. Anthropométrie de 1023 Coniagui, Bassari, Badyaranké et Fulakunda de Guinée française. Bulletins et Mémoires de la Société d'anthropologie de Paris, X Series 1: 99–136.

Lewis, M. Paul, Gary F. Simons, and Charles D. Fennig (eds.). 2015. Ethnologue: Languages of the World, Eighteenth edition. Dallas, Texas: SIL International. Online version: http://www.ethnologue.com.

Lombard J (1960) La vie politique dans une ancienne société de type féodal: Les Bariba du Dahomey. Cahiers d'Études Africaines 1: 5–45.

Machado AJM (1972) Gentes de Catió. Geographica 8: 3–32.

Maclaud C (1907) Notes anthropologiques sur les Diola de la Casamance. L'Anthropologie 18: 69–98.

Mark P (1978) Urban Migration, Cash Cropping, and Calamity: The Spread of Islam among the Diola of Boulouf (Senegal), 1900-1940. African Studies Review 21: 1–14.

Martin Y (2001) Foreskin lament for babies. The Press, Canterbury, New Zealand, January 13, 2001.

McCaskie TC (1981) State and Society, Marriage and Adultery: Some Considerations Towards a Social History of Pre-Colonial Asante. J Afr Hist 22: 477–494.

Meireles AM (1960) Mutilações étnicas nos Manjacos. Bissau: Centro de Estudos da Guiné Portuguesa.

Mendes FA (1948) Vida material dos Brâmes. Boletim Cultural da Guiné Portuguesa 3: 81–113.

Menjaud H (1932) Documents ethnographiques sur le Gourma. Journal de la Société des Africanistes 2: 35–47.

Mercier P (1947) Marques du statut individuel chez les Somba. In: Conferência Internacional dos Africanistas Orientais, Bissau, 1947, Volume V. Lisboa: Junta de Investigações Coloniais.

Mercier P, Balandier G (1952) Les pêcheurs Lebou du Sénégal, Particularisme et évolution. Saint Louis, Sénégal: Centre IFAN-Sénégal.

Migeod FWH (1925) A view of the colony of Sierra Leone. J Afr Soc 25: 1–9.

Miner H (1942) Songhoi Circumcision. Amer Anthropol, New Series 44: 621–637.

Moreau RL (1964) Les marabouts de Dori. Archives de Sociologie des Religions 9: 113–134.

Murdock GP (1967) Ethnographic Atlas. Pittsburgh: Univ of Pittsburgh Press.

Niang CI, Boiro H (2007) ‘‘You Can Also Cut My Finger!’’: Social Construction of Male Circumcision in West Africa, A Case Study of Senegal and Guinea-Bissau. Reprod Health Matters 15: 22–32.

Niangoran-Bouah G (1964) La division du temps et le calandrier rituel des peuples lagunaires de Côte d’Ivoire. Paris: Institut d’Ethnologie.

Nogueira A (1947) Monografia da Tribo Banhum. Boletim Cultural da Guiné Portuguesa 2: 973–1008.

Paroisse G (1896) Notes sur les peuplades autochtones de la Guinée Française (Rivières du Sud). L'Anthropologie 7: 428–442.

Parsons T (1999) The British Imperial Century, 1815-1914: A World History Perspective. New York: Rowman & Littlefield.

Paulme D (1947) L'initiation des filles en pays Kissi (Haute-Guinée). In: Conferência Internacional dos Africanistas Orientais, Bissau, 1947, Volume V. Lisboa: Junta de Investigações Coloniais.

Paulme D (1962) Une societé de Côte d’Ivoire hier et aujourd’hui: les Bété. Paris: Mouton & Co.

Paulme D, editor (1971) Classes et associations d'age en Afrique de l'Ouest. Paris: Librairie Plon.

Pélissier R (1989) História da Guiné: Portugueses e Africanos na Senegâmbia, 1841–1936. Lisboa: Editorial Estampa.

Peperti R (1945) La circoncision et l'excision chez les Tankambas de la subdivision de Tanguieta. Conférence Internationale des Africanistes de l'Ouest, Dakar, January 19-25, 1945.

Pépin J, Plamondon M, Alves AC, Beaudet M, Labbé AC (2006) Parenteral transmission during excision and treatment of tuberculosis and trypanosomiasis may be responsible for the HIV-2 epidemic in Guinea-Bissau. AIDS 20: 1303–1311.

Picard F (1910) Moeurs et coutumes des indigènes de la boucle du Niger. Bulletins et Mémoires de la Société d'anthropologie de Paris, XI Series 1: 422–443.

Pison G, Le Guenno L, Lagarde E, Enel C, Seck C (1993) Seasonal Migration: a Risk Factor for HIV Infection in Rural Senegal. J Acquir Immune Def Syndr 6: 196–200.

Pobee JS, Mends EH (1977) Social Change and African Traditional Religion. Sociological Analysis 38: 1–12.

Porter AT (1953) Religious affiliation in Freetown, Sierra Leone. Africa 23: 3–14.

Prasse KG (1995) The Tuaregs: The Blue People. Museum Tusculanum Press. Copenhagen: Museum Tusculanum Press, University of Copenhagen.

Quintino FC (1969) Os povos da Guiné. Boletim Cultural da Guiné Portuguesa 24: 861–916.

Ruelle E (1904) Notes anthropologiques, ethnographiques et sociologiques sur quelques populations noires du 2éme Territoire Militaire de l'Afrique Occidentale Française. L'Anthropologie 15: 519–561; 657–703.

Samwini N (2006) The Muslim Resurgence in Ghana Since 1950: Its Effects Upon Muslims and Muslim-Christian Relations. Münster: LIT-Verlag.

Sanderson IT (1935) An Expedition to the British Cameroons. The Geographical Journal 85: 113–140.

Sanneh L (1984) Prelude to African Christian Independency: The Afro-American Factor in African Christianity. The Harvard Theological Review 77: 1–32.

Sanogo M, Pageard R (1964) Notes sur les coutumes des marka de Lanfiéra. Journal de la Société des Africanistes 34: 306–310.

Sarpong PK (1967) The sacred stools of Ashanti. Anthropos 62: 1–60.

Scantamburlo L (1991) Etnologia dos Bijagós da ilha de Bubaque. Lisboa: Instituto de Investigação Científica Tropical; Bissau: Instituto Nacional de Estudos e Pesquisa.

Schwab C (1947) Tribes of the Liberian hinterland: Report of the Peabody Museum expedition to Liberia. Cambridge, MA: Peabody Museum of American Archaeology and Ethnology.

Siegmann W (1969) Ethnographic Survey of Southeastern Liberia: Report on the Bassa. Monrovia: Tubman Center of African Culture.

Stahl AB (1991) Ethnic Style and Ethnic Boundaries: A Diachronic Case Study from West-Central Ghana. Ethnohistory 38: 250–275.

Staude W (1962) La structure de la chefferie chez les Kouroumba de Louroum (Haute-Volta septentrionale). Un essai. Anthropos 57: 757–778.

Strong RP, editor (1930) The African Republic of Liberia and the Belgian Congo, Based on the Observations Made and Material Collected During the Harvard African Expedition, 1926–27. Cambridge, MA: Harvard University Press.

Tauxier L (1931) Les Dorhosié et Dorhosié-Finng du cercle do Bobo-Dioulasso (Soudan français). Journal de la Société des Africanistes 1: 61–86.

Tauxier L (1933) Les Gouin et les Tourouka, résidence de Banfora, cercle de Bobo-Dioulasso. Étude ethnologique, suivie d'un double vocabulaire. Journal de la Société des Africanistes 3: 77–128.

Teixeira da Mota A (1947) Inquérito etnográfico – organizado pelo Governo da Colónia no ano de 1946. Bissau: Governo da Colónia da Guiné Portuguesa.

Tremearne AJN (1913) Hausa Superstitions and Customs: an Introduction to the Folk-Lore and the Folk. London: John Bale, Sons, and Danielsson.

UNAIDS (2007) Male circumcision: Global trends and determinants of prevalence, safety and acceptability. Geneva: WHO and UNAIDS.

Vallois HV (1941) Recherches anthropologiques sur les Peuls et divers Noirs de l'Afrique occidentale d'après les mensurations de M. Leca (Mission Labouret, 1932). Bulletins et Mémoires de la Société d'anthropologie de Paris, IX Series 2: 20–74.

Van Howe RS (1999) Circumcision and HIV infection: review of the literature and meta-analysis. Int J STD & AIDS 10: 8–16.

Villamur R (1903) Notre Colonie de la Côte d'Ivoire. Paris: Augustin Challamel.

Wilson D, de Beyer J (2008) Male Circumcision: Evidence and Implications. Washington DC: World Bank Global HIV/AIDS Program.

Zaborowski M (1896) La circoncision: ses origines et sa répartition en Afrique et a Madagascar. L'Anthropologie 7: 653–675.

Zetterström K (1969) Ethnographic Survey of Southeastern Liberia: Preliminary Report on the Kru. Monrovia: Tubman Center of African Culture.

Part III – References of the Datasets of tables of ethnic partition of population of cities and regions

Administração do Concelho de Bissau (1970) Resumo final das populações do Concelho de Bissau por regulados. Bissau: Imprensa Nacional da Guiné.

Agência Geral do Ultramar (1966) Cabo Verde: pequena monografia. Lisboa: Agência Geral do Ultramar.

Agier M (1983) Commerce et sociabilité: les négociants soudanais du quartier zongo de Lomé (Togo). Paris: Éditions de l'ORSTOM.

Amaral I (1964) Santiago de Cabo Verde: a Terra e os Homens. Universidade de Lisboa, thesis dissertation, 1964.

Antoine P, Dubresson A, Manou-Savina A (1987) Abidjan «côté cours». Paris: Éditions Karthala and Éditions de l'ORSTOM.

Agência Geral das Colónias (1929) Guiné. Boletim da Agência Geral das Colónias 44, separata.

Banton M (1956) Adaptation and integration in the social system of Temne immigrants in Freetown. J Int Afr Inst 26: 354–368.

Banton M (1957) West African city: a study of tribal life in Freetown. London: Oxford University Press.

Bernus S (1969) Particularismes ethniques en milieu urbain: l'exemple de Niamey. Paris: Institut d'Ethnologie, Musée de l'Homme.

Carreira A (1962) Guiné Portuguesa: população autóctone segundo os recenseamentos para fins fiscais. Bissau: Imprensa Portuguesa.

Carvalho Viegas LA (1936–40) Guiné Portuguesa [Vol I: 1936; Vol II: 1939; Vol III: 1939–40]. Lisboa: Ministério do Ultramar e Colónia Portuguesa da Guiné.

Coleman JS (1958) Nigeria: background to nationalism. Berkeley: University of California Press.

Collomb H, Ayats H (1962) Les migrations au Sénégal: étude psychopathologique. Cahiers d'Études Africaines 2: 570–597.

Côte d'Ivoire (1934) Colonie de la Côte d'Ivoire: Rapport Politique. Series 2G34, Document 7, Archives Nationales d'Outre Mer, Aix-en-Provence, France.

Côte d'Ivoire (1960) Recensement d'Abidjan 1955: résultats définitifs. Abidjan: Ministère des Finances, des Affaires Économiques et du Plan, Direction de la Statistique et des Études Économiques et Démographiques.

Delafosse M (1912) Haut-Sénégal-Niger: le pays, les peuples, les langues. Paris: Larose.

Delval R (1980) Les Musulmans au Togo. Paris: Centre de Hautes Études sur l'Afrique et l'Asie Modernes.

Duarte Silva AE (1997) A independência da Guiné-Bissau e a descolonização portuguesa. Porto: Edições Afrontamento.

Dureau F (1987) Migration et urbanization: le cas de la Côte d'Ivoire. Paris: Éditions de l'ORSTOM.

Fraenkel M (1964) Tribe and Class in Monrovia. London: Oxford Univ Press.

Froelich JC (1955) Carte des populations de l'Afrique Noire. Paris: Sécretariat Général du Gouvernement, Direction de la Documentation.

Gouvernement Général de l'Afrique Occidentale Française (1931) La Côte d'Ivoire. Exposition Coloniale Internationale. Paris: Société d'Éditions Géographiques, Maritimes et Coloniales.

Goerg O (1990) La genèse du peuplement de Conakry. Cahiers d'Études Africaines 31: 73–99.

Goerg O (2006) Chieftainships between Past and Present: From City to Suburb and Back in Colonial Conakry, 1890s-1950s. Africa Today 52: 3–27.

Gold Coast (1950) Census of population 1948: report and tables. London: Government of the Gold Coast.

Harvey ME (1971) Social Change and Ethnic Relocation in Developing Africa: the Sierra Leone Example. Geografiska Annaler Series B 53:94–106.

Hesseling G (1992) Pratiques foncières à l'ombre du droit: l'application du droit foncier urbain à Ziguinchor, Sénégal. Leiden: African Studies Centre.

Jarret HR (1951) Bathurst: port of the Gambia river. Geography 36: 98–107.

Junta de Investigações do Ultramar (1950) Província da Guiné: Censo da População. Lisboa: Centro de Estudos Políticos e Sociais.

Lahmeyer J (2006) Population Statistics: historical demography of all countries, their divisions and towns. Available: http://www.populstat.info.

Le Pape M (1993) L'attraction urbaine: soixante-cinq ans d'observations sur Abidjan. Cahiers Sci Hum 29: 333–348.

Marguerat Y (1982) Des ethnies et des villes: Analyse des migrations vers les villes de Côte d'Ivoire. Cahiers de l'ORSTOM, Série Sci Hum 18: 303–340.

Meillassoux C (1965) The Social Structure of Modern Bamako. Africa: J Int Afr Inst 35: 125–142.

Ministério dos Negócios Estrangeiros (1978) IX Recenseamento Geral da População, 1960, Província da Guiné. Lisboa: Ministério dos Negócios Estrangeiros, Serviços de Administração Civil, Secção de Estatística.

Ndione B (2008) Territoires urbains et réseaux sociaux : les processus de migration internationale dans les quartiers de la ville sénégalaise de Kaolack. African Diaspora 1: 110–133.

Pauvert JC (1960) L'évolution politique des Ewé. Cahiers d'Études Africaines 1: 161–192.

Pélissier R (1989) História da Guiné: Portugueses e Africanos na Senegâmbia, 1841–1936. Lisboa: Editorial Estampa.

Sénégal (1934) Circonscription de Dakar et Dépendances. Services Sanitaires et Médicaux. Rapport Annuel 1934. Dakar: Services Sanitaires et Médicaux. Archives of Institut de Médecine Tropicale du Service de Santé des Armées, box 26.

Sénégal (1950–52) Hôpital Central Africain. Rapport Annuel [1950 through 1952]. Dakar: Service de Santé. Archives of Institut de Médecine Tropicale du Service de Santé des Armées, box 32.

Sénégal (1955) Service d'Hygiène de Dakar. Rapport Annuel 1955. Dakar: Service d'Hygiène. Archives of Institut de Médecine Tropicale du Service de Santé des Armées, box 36.

Skinner EP (1974) African Urban Life: the Transformation of Ouagadougou. Princeton: Princeton University Press.

Sotindjo SD (2009) Cotonou: l'explosion d'une capitale économique (1945–1985). Paris: L'Harmattan.

Tardits C (1958) Porto-Novo: les nouvelles générations africaines entre leurs traditions et l'Occident. Paris: Mouton & Co.

Tardits C (1962) Réflexions sur le problème de la scolarisation des filles au Dahomey. Cahiers d'Études Africaines 3: 266–281.

Wilkins A, Hayes R, Alonso P, Baldeh S, Berry N, et al. (1991) Risk factors for HIV-2 infection in The Gambia. AIDS 5: 1127–1132.

Part IV – Personal Communications

Marc Augé (École des Hautes Études en Sciences Sociales, France), email communication, January 2009: The Lagunaire peoples of Côte d'Ivoire (Ebrié, Mbato, Abouré, Aladian, Avikam, Adioukrou) were not traditionally circumcised.

Daniel Elwood Dunn (Alfred Walter Negley Professor of Political Science, University of the South, Sewanee, USA), email communication, March 2009: at about 1950, most boys of mixed immigrant[Americo-Liberian]-indigenous heritage were probably being circumcised, based on his own and his peers experience.

Francisco Henriques da Silva (Historian, participant in the Guinea-Bissau war in 1968–1970, Ambassador of Portugal in Guinea-Bissau in 1997–1999), January 2015: Cape Verdeans who were in Guinea-Bissau during the independence war did not practice male circumcision.
