# Supplementary material for: Male Circumcision and the Epidemic Emergence of HIV-2 in West Africa
Source: PLoS One. 2016 Dec 7;11(12):e0166805. doi: 10.1371/journal.pone.0166805 (PMC5142780; doi:10.1371/journal.pone.0166805)
Supplement: S1 Protocol — (ZIP) [file PC_DanielElwoodDunn.pdf › PC_DanielElwoodDunn.pdf]

X-AntiVirus: PTMail-AV 0.3-0.90.0
X-Scan-Status: AV clean (0.01407 seconds);
AS clean (0.03701 seconds);
Date: Wed, 18 Mar 2009 10:23:42 -0500
To: Joao Sousa <j.d.sousa@sapo.pt>
From: Elwood Dunn <edunn@sewanee.edu>
Subject: Re: Hello Dr Daniel Elwood Dunn
X-Synonym: Copied by Synonym (http://www.modulo.ro/synonym) to:
facstaffar@emailarchive.sewanee.edu

I would say: " most boys of mixed immigrant-indigenous heritage were already PROBABLY being circumcised."

No problem with citing me as a source.

Would appreciate seeing product of your work.

> Dear Dr Elwood Dunn,
>
> Yes, I have information about all the indigenous ethnic groups. I made my question below because I was not sure that all the peers that you mentioned were also of immigrant (or mixed immigrant-indigenous) heritage. But now that's clear. So, I can conclude that about 60 years ago, most boys of mixed immigrant-indigenous heritage were already being circumcised?
>
> And if you agree with my conclusion above, can I cite this assertion as being a Personal Communication from you in my writings?
>
> PS- I will be glad to present you my work.
>
> Best
> João
>
> At 14:38 18-03-2009, you wrote:
>> I am now 67 years old, so it was 60 years ago. Yes, I would say my mixed heritage peers.
>> I thought you had scientific information on all the indigenous groups.
>>
>>> Dear Dr Elwood Dunn,
>>>
>>> Thank you for sharing with me your experience of circumcision, which is helpful for me. Could I just ask you which year was it? And when you say that many of your peers were also circumcised, you mean those of mixed immigrant and indigenous heritage like you?

--
D. Elwood Dunn
The Alfred Walter Negley Professor of Political Science
Sewanee:University of the South
Sewanee TN 37383
Phone: 931/598-1547
Fax: 931/598-1145
E-mail: edunn@sewanee.edu
