# Supplementary material for: Male Circumcision and the Epidemic Emergence of HIV-2 in West Africa
Source: PLoS One. 2016 Dec 7;11(12):e0166805. doi: 10.1371/journal.pone.0166805 (PMC5142780; doi:10.1371/journal.pone.0166805)
Supplement: S1 Protocol — (ZIP) [file PC_FranciscoHenriquesSilva.pdf › PC_FranciscoHenriquesSilva.pdf]

X-KULeuven-Envelope-From: fhsilva60@gmail.com
X-Spam-Status: not spam, SpamAssassin (not cached, score=-0.798, required 5,
autolearn=disabled, DKIM_SIGNED 0.10, DKIM_VALID -0.10,
DKIM_VALID_AU -0.10, FREEMAIL_FROM 0.00, RCVD_IN_DNSWL_LOW -0.70,
URIBL_BLOCKED 0.00)
X-KULeuven-Scanned: Found to be clean
X-KULeuven-ID: 6CFC813802C.A44D2
X-KULeuven-Information: Katholieke Universiteit Leuven
DKIM-Signature: v=1; a=rsa-sha256; c=relaxed/relaxed;
d=gmail.com; s=20120113;
h=mime-version:subject:from:in-reply-to:date
:content-transfer-encoding:message-id:references:to;
bh=q3DdCYubti0RQYc4eQvpaNJlqRdRU2Ai5ORwXRTPZ1A=;
b=wr0n3sDUQcPlciMEYcI7DhNdASy0RjCZLG+ct4XfdXsjQzuV++WVlMRAMpp08VXk41
C6u7aEZg493qkf3Mdt+6l8ffnUG/hlyvmKSdppRWMB/bn+qHTo4T/xsJS3mqIRd9/kfY
vDIOuByyoepCNa9UXuL6yvG7wPZ86s5rOji5NqXDvmwF928t4kezVnf5Y/1zK1nYX7YF
cFGueE2UYcDXN+i2cV80df9FvLWaZSKh/9Hyry18INwsHTSXyf5KEgKgNtEQ1m2lX6U1
yIn8Ojjvfqd+vgPrvTfO5jaWCl9nR6poScb1CTnROhIfxMlz9B8xBMGk3PieyeiV7DRE
+Jiw==
X-Google-DKIM-Signature: v=1; a=rsa-sha256; c=relaxed/relaxed;
d=1e100.net; s=20130820;
h=x-gm-message-state:mime-version:subject:from:in-reply-to:date
:content-transfer-encoding:message-id:references:to;
bh=q3DdCYubti0RQYc4eQvpaNJlqRdRU2Ai5ORwXRTPZ1A=;
b=J8S2fzDDOKWkuQvUKcnbQC0z36GEKmQ7LLdiVSvzWX/82/Wn3tjB6pLcmtfE6O1Il5
hhaGB0zbsKFUG0hUQ3Pmu0tkeJt25E+MAuZOQ0dyEIN1g4cHB7cMmD2NM6rUSC3XDDLI
T6GzQdPoz7R5XMT3RIda/VahbB6sJ7IL4K/1TyKIJsYF0OY2zhb4lkl5CfdWlSKmj/Xj
Qh9DMjoCdjLnkvHRHR+1g6u16EegyMNGhYoTDbc5zxCOzN92goPurfF8B2TXhcFzbxzK
QWzZGHIu+i3Iiempur9J4gEaSxB/Q7d2FAiJigkemh0z5it+pVwr0Fbu2NrE3UPX/S1b
DZIQ==
X-Gm-Message-State:
ALyK8tKYeOhyPyvEfb+6f2J/woKnKM5B3KypguzY2b4ZWXhk1e2MNdfdm+8IGBZjNYw+wg==
X-Received: by 10.28.87.3 with SMTP id l3mr30475456wmb.71.1468449199748;
Wed, 13 Jul 2016 15:33:19 -0700 (PDT)
Subject: Re: Cape Verdeans in Guinea-Bissau during the independance war
From: "Francisco H. da Silva" <fhsilva60@gmail.com>
Date: Wed, 13 Jul 2016 23:33:16 +0100
To: Joao Sousa <joao.sousa@kuleuven.be>
X-Mailer: Apple Mail (2.3124)
X-MS-Exchange-Organization-Network-Message-Id: 98c784df-e4da-4bcd-2df5-08d3ab6db3a4
X-MS-Exchange-Organization-AuthSource: ICTS-S-EXMBX1.luna.kuleuven.be
X-MS-Exchange-Organization-AuthAs: Anonymous

Dear Dr. João Sousa,

Regarding our previous contact I can confirm in writing the information I have provided you then.

In fact, the Cape Verdeans being Christians, almost exclusively of Catholic extraction, or even if they were atheists, their cultural roots were the same, they did not practice male circumcision at all. I am not aware that a few could have practiced it to be better accepted by the Guineans. That's basically a myth. In spite of the fact that local Guinean tribes practice the "fanado" (male circumcision) I don't know of a single case of a Cape Verdean which has undergone that procedure, that would necessarily imply a tribal cultural tradition, heavily charged with symbolism. The Cape Verdeans were "assimilados", in the old Portuguese legal colonial framework, so they would be considered as whites. They were, of course, devoid of any tribal background. This would apply to those who have joined the guerrilla warfare.

You can quote me freely on this as it is my opinion having lived through the colonial war 1968-1970 and later on having served as ambassador of Portugal to Guinea-Bissau (1997-1999).

Thank you for your interest in this matter

Best regards

Francisco Henriques da Silva

´

> No dia 13/07/2016, às 23:02, Joao Sousa <joao.sousa@kuleuven.be> escreveu:

>
> Dear Dr Francisco Henriques da Silva,
>
> I thank you very much for your information. Do you give me permission to cite it as a personal communication from you (of course identifying you as the source) in my articles? When answering, please include the text below.
>
> Thank you
> Best regards
> João Sousa
>
> At 22:36 13/07/2016, you wrote:
>> Estimado dr. João Dinis Sousa,
>>
>> The Cape Verdeans being Christians, almost exclusively of Catholic extraction, or even if they were atheists, their cultural roots were the same, so they did not practice male circumcision at all. I am not aware that a few could have practiced it to be better accepted by the Guineans. That's basically a myth. In spite of the fact that local Guinean tribes practice the "fanado" (male circumcision) I don't know of a single case of a Cape Verdean which has undergone that procedure, that would necessarily imply a tribal cultural tradition, heavily charged with symbolism. The Cape Verdeans were "assimilados", in the old Portuguese legal colonial framework, so they would be considered as whites. They were devoid of any tribal background. This would apply to those who have joined the guerrilla warfare.â?
>> I hope this information was useful.
>>
>> Best regards
>>
>> Francisco Henriques da Silva
>>
>
>
>

>
> "A common man marvels at uncommon things; a wise man marvels at the commonplace" - Confucius
>
>
> João Dinis Sousa
> Katholieke Universiteit Leuven
> Laboratory for Clinical and Epidemiological Virology
> Rega Institute for Medical Research
> Minderbroedersstraat 10
> B-3000 Leuven, Belgium
> Phone: +32-16-332160
> Fax: +32-16-332131
> E-mail: joao.sousa@kuleuven.be
>
